# Combined hybridization capture and shotgun sequencing for ancient DNA analysis of extinct wild and domestic dromedary camel

**DOI:** 10.1111/1755-0998.12551

**Published:** 2016-08-01

**Authors:** Elmira Mohandesan, Camilla F. Speller, Joris Peters, Hans‐Peter Uerpmann, Margarethe Uerpmann, Bea De Cupere, Michael Hofreiter, Pamela A. Burger

**Affiliations:** ^1^ Research Institute of Wildlife EcologyVetmeduni ViennaSavoyenstraße 11160ViennaAustria; ^2^ Institute of Population GeneticsVetmeduni ViennaVeterinärplatz 11210ViennaAustria; ^3^ BioArChDepartment of ArchaeologyUniversity of YorkWentworth WayYorkYO10 5DDUK; ^4^ Department of Veterinary SciencesInstitute of PalaeoanatomyDomestication Research and the History of Veterinary Medicine Ludwig‐Maximilians‐Universität München (LMU Munich)80539MunichGermany; ^5^ Staatliche Naturwissenschaftliche Sammlungen BayernsBavarian State Collection of Anthropology and Palaeoanatomy80333MunichGermany; ^6^ Abteilung ArchäozoologieInstitut für Naturwissenschaftliche ArchäologieEberhard‐Karls‐Universität TübingenRümelinstrasse 237207TübingenGermany; ^7^ Royal Belgian Institute of Natural SciencesVautierstraat 29B‐1000BrusselsBelgium; ^8^ Evolutionary and Adaptive GenomicsDepartment of Mathematics and Natural SciencesInstitute for Biochemistry and BiologyUniversity of PotsdamKarl‐Liebknecht‐Street 24‐25Potsdam14476Germany

**Keywords:** ancient DNA, *Camelus dromedarius*, capture enrichment, degraded DNA, mitochondrial genome (mtDNA), next‐generation sequencing

## Abstract

The performance of hybridization capture combined with next‐generation sequencing (NGS) has seen limited investigation with samples from hot and arid regions until now. We applied hybridization capture and shotgun sequencing to recover DNA sequences from bone specimens of ancient‐domestic dromedary (*Camelus dromedarius*) and its extinct ancestor, the wild dromedary from Jordan, Syria, Turkey and the Arabian Peninsula, respectively. Our results show that hybridization capture increased the percentage of mitochondrial DNA (mtDNA) recovery by an average 187‐fold and in some cases yielded virtually complete mitochondrial (mt) genomes at multifold coverage in a single capture experiment. Furthermore, we tested the effect of hybridization temperature and time by using a touchdown approach on a limited number of samples. We observed no significant difference in the number of unique dromedary mtDNA reads retrieved with the standard capture compared to the touchdown method. In total, we obtained 14 partial mitochondrial genomes from ancient‐domestic dromedaries with 17–95% length coverage and 1.27–47.1‐fold read depths for the covered regions. Using whole‐genome shotgun sequencing, we successfully recovered endogenous dromedary nuclear DNA (nuDNA) from domestic and wild dromedary specimens with 1–1.06‐fold read depths for covered regions. Our results highlight that despite recent methodological advances, obtaining ancient DNA (aDNA) from specimens recovered from hot, arid environments is still problematic. Hybridization protocols require specific optimization, and samples at the limit of DNA preservation need multiple replications of DNA extraction and hybridization capture as has been shown previously for Middle Pleistocene specimens.

## Introduction

The pioneering world of next‐generation sequencing (NGS) (Margulies *et al*. 2005; Millar *et al*. 2008; Shendure & Ji 2008) has advanced the field of aDNA tremendously, from sequencing short fragments of mtDNA (Higuchi *et al*. 1984) to generating data sets of genome scale from extant and extinct species (Green *et al*. 2010; Reich *et al*. 2010; Orlando *et al*. 2011, 2013; Meyer *et al*. 2012; Prüfer *et al*. 2014; Rasmussen *et al*. 2014). Although whole ancient genomes are becoming more readily accessible, mitochondrial genomes (mitogenomes) are still the marker of choice in aDNA studies dealing with samples with very poor DNA preservation (Dabney *et al*. 2013; Meyer *et al*. 2014), or when comparing mitochondrial diversity between ancient and modern populations (Thalmann *et al*. 2013; Zhang *et al*. 2013; Almathen *et al*. 2016). Despite recent methodological progress, aDNA research is still fraught with technical complications, such as low template quantities, high fragmentation, miscoding lesions (Stiller *et al*. 2006; Briggs *et al*. 2007, 2010; Brotherton *et al*. 2007; Sawyer *et al*. 2012) and contamination with modern DNA (Green *et al*. 2006; Surakka *et al*. 2010; Rasmussen *et al*. 2011). Only in few cases, such as permafrost samples (Palkopoulou *et al*. 2015), rare cave findings (Reich *et al*. 2010; Prüfer *et al*. 2014) or when sampling the petrous bone of the cranium (Gamba *et al*. 2014; Pinhasi *et al*. 2015), a high ratio of endogenous DNA (4–85%) vs. environmental and contaminant DNA has been reported. Moreover, the rate of DNA integrity is negatively correlated with the ambient temperature to which the samples were exposed (Smith *et al*. 2001; Allentoft *et al*. 2012; Hofreiter *et al*. 2015). While poor DNA preservation from palaeontological samples collected in arid regions poses significant technical challenges (Paijmans *et al*. 2015), aDNA sequences have occasionally been reported from arid regions and contributed significantly to understanding prehistoric events (e.g. Orlando *et al*. 2006; Bollongino *et al*. 2013; Meiri *et al*. 2013; Fernández *et al*. 2014; Almathen *et al*. 2016). In this study, we focused on archaeological samples from wild and domestic dromedaries, a species typically associated with hot and arid regions.

The single‐humped dromedary (*Camelus dromedarius*) is the most numerous and widespread domestic camel species inhabiting northern and eastern Africa, the Arabian Peninsula and southwest Asia; a large feral population exists in Australia (Köhler‐Rollefson 1991; Spencer & Woolnough 2010). Dromedaries are bred for multiple purposes including meat, milk, wool, transportation and sport (Bulliet 1990; Grigson 2012). They are particularly well adapted to hot, desert conditions and show a variety of biological and physiological characteristics of evolutionary, economic and medical importance (Wu *et al*. 2014). Zooarchaeological research suggests that the domestication of dromedaries (*C. dromedarius*) occurred between 1500 and 1000 BCE (before the common era) on the southeast coast of the Arabian Peninsula (Rowley‐Conwy 1988; Uerpmann & Uerpmann 2002; Iamoni 2009; Grigson 2012; Uerpmann & Uerpmann 2012; Magee 2015). This has recently been confirmed by phylogenetic and phylogeographical analyses of modern global dromedary populations, including aDNA analysis of wild dromedaries (Almathen *et al*. 2016), which likely became extinct in the early first millennium CE (Uerpmann & Uerpmann 2002; von den Driesch *et al*. 2008; Uerpmann & Uerpmann 2012; Grigson 2014).

The remains of a single large‐sized Late Pleistocene camel individual recovered from the Site 1040 near Wadi Halfa were first evaluated by Gautier (1966), who assigned them to *Camelus thomasi*, the giant North African camel. Based on a limited number of comparative specimens and few metrical data, the author at that time concluded that the Site 1040 specimen exhibited close relationship to the two‐humped domestic camel *Camelus bactrianus*. Following this study, Peters (1998) revisited the same assemblage by using a much larger set of comparative specimens and drawing on the work of Steiger (1990). This revision concluded that all specimens available for restudy, that is distal humerus, distal radius ulna, distal tibia and calcaneus, exhibited features characteristic not of the two‐humped but of the one‐humped camel *C. dromedarius*. Towards the end of the Pleistocene, *C. thomasi* likely disappeared from Africa, given its absence in archaeological sites, natural deposits and rock art dating to the Holocene. The proximity of northeast Africa and the Arabian Peninsula opens up the possibility that either *C. thomasi* or a closely related form survived in southwest Asia, giving rise in to the wild ancestor of domestic population at the transition of the Late Bronze to the Iron Age.

The study of aDNA thus presents a unique opportunity to explore the genetic make‐up and variation in a wild progenitor population prior to the species’ domestication. In other livestock species, an increasing number of genetic studies have taken advantage of ancient and historical samples from both extant and extinct species (Elbaum *et al*. 2006; Amaral *et al*. 2011; Cai *et al*. 2011; Kimura *et al*. 2011; Zhang *et al*. 2013; Girdland Flink *et al*. 2014; Schubert *et al*. 2014) to investigate the historical domestication process. However, no genetic data from archaeological dromedary specimens have been available until recently (Almathen *et al*. 2016). This could be due to the general rarity of *C. dromedarius* specimens in archaeological contexts, even within the current and historical geographical distributions of dromedaries, and the challenging task of obtaining DNA from archaeological remains in desert regions.

In this study, we explore two methodological strategies to recover mitochondrial genomes from ancient dromedary specimens: (i) double‐ or single‐stranded DNA library (DSL or SSL) preparation (Meyer & Kircher 2010; Gansauge & Meyer 2013; Fortes & Paijmans 2015) followed by hybridization enrichment (Briggs *et al*. 2009; Maricic *et al*. 2010; Fu *et al*. 2013) and NGS sequencing and (ii) DSL preparation followed by whole‐genome shotgun sequencing. We describe the efficiency of the enrichment method, when applied to aDNA libraries with variable levels of endogenous DNA. We also compare the effect of hybridization condition on recovering the captured targets after the hybridization step in two different enrichment methods. This study highlights one of the few successful recoveries of DNA sequences from specimens excavated in hot and arid environments.

## Materials and methods

### Ancient‐domestic and wild dromedary samples

**Figure 1:**
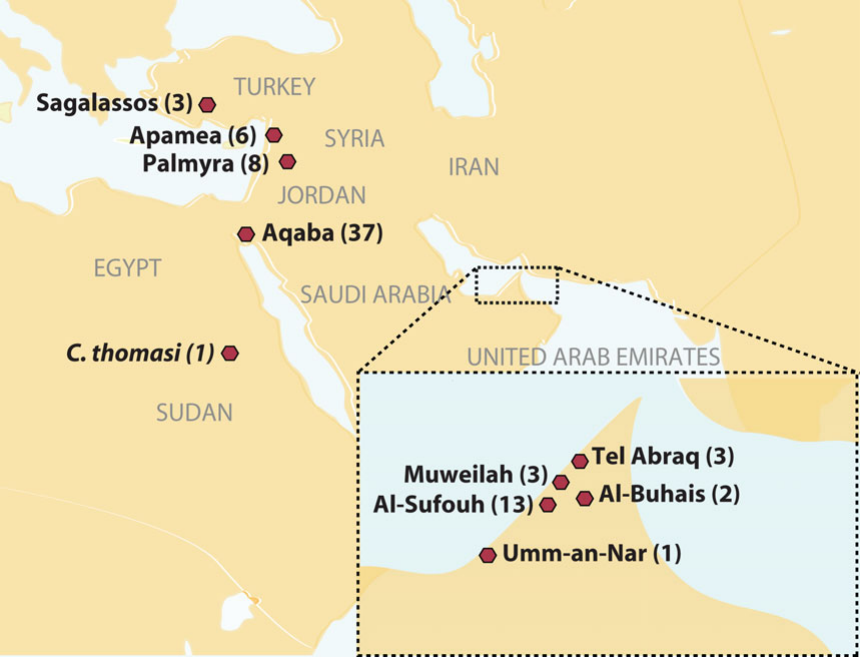
Geographical locations of the ancient‐domestic dromedary, its extinct ancestor the wild dromedary and the giant camel (*Camelus thomasi*) used in this study. [Colour figure can be viewed at wileyonlinelibrary.com]

We analysed 54 ancient‐domestic dromedary samples (100 BCE – 1870 CE) from excavation sites in Sagalassos, Turkey (Early Byzantine: 450–700 CE); Apamea, Syria (Early Byzantine: 400–600 CE); Palmyra, Syria (100 BCE – 300 CE); and Aqaba, Jordan (Ottoman: 1456–1870 CE, Mamluk: 1260–1456 CE). We also analysed 22 wild dromedary specimens (5000–1130 BCE) from archaeological sites of Al Sufouh‐2 (Wadi Suq Middle Bronze Age *ca*. 2000–1600 BCE); Tell Abraq (Late Bronze – Iron Age: 1260–500 BCE); Muweilah (older than 1000–586 BCE); Umm an‐Nar (Early Bronze Age: 3000–2000 BCE); and Al‐Buhais 18 (5000–4000 BCE) in the United Arab Emirates (UAE). In addition, we analysed one Upper Palaeolithic wild giant camel sample (*Camelus thomasi*) found below sediments dated to *ca*. 20 000 BCE and collected during the Combined Nubian Prehistory and Geological Campaign in the early 1960s at Site 1040, located in the northern Sudanese Nile Valley close to Wadi Halfa, near the boundary with Egypt. The description of the samples and their geographical location are detailed in Table S1 (Supporting information) and Fig. 1.

### Holocene climate change in regions of sample collection

After the initial warming at the end of the Ice Age (around 10 000 BCE), the climate in the Middle East began to change from cooler and moister (~4000 BCE) to warmer and more arid (~3000 BCE), reaching today's condition only at the very beginning of the Iron Age (~1200 BCE) (Preston *et al*. 2015; Hume *et al*. 2016), which according to present data coincides with the early domestication stages of the dromedary. Nevertheless, there is no evidence that the aridification caused the domestication of camels in this region. It may, however, have increased the value of tamed camels, which would have become more useful during times of drought. Although the climatic and environmental conditions from where the samples were collected varied to some extent during the Holocene, they allowed for the existence of dromedaries in all the respective areas.

### Ancient DNA extraction

The bone samples were prepared in a dedicated and highly contained aDNA laboratory at the Palaeogenetic Core Facility of the ArchaeoBioCenter at the LMU Munich, Germany, with appropriate contamination precautions in place (Knapp *et al*. 2012). For each sample, approximately 200–250 mg of bone powder was used for DNA extraction. Two independent DNA extractions in the presence of extraction blanks (one blank per six extractions) were conducted following a silica‐based extraction protocol (Rohland & Hofreiter 2007; Rohland *et al*. 2010). DNA was eluted in 50 μL TET buffer and stored at −20 °C. In addition, we extracted DNA from a subset of wild dromedaries (six samples) and one ancient giant camel (*C. thomasi*) in the presence of one extraction blank, using the Dabney *et al*. (2013) DNA extraction protocol. In this method, we used approximately 120–125 mg of bone powder and the final DNA extracts were eluted in 25 μL TET. The DNA extracts obtained by applying the Rohland *et al*.'s (2010) protocol were used for double‐stranded DNA library preparation (DSL) (Meyer & Kircher 2010), while the DNA extracts following Dabney *et al*. (2013) were used for single‐stranded library (SSL) preparation (Gansauge & Meyer 2013). To recover greater quantities of short DNA fragments, we combined Dabney *et al*.'s (2013) DNA extraction and SSL methods (Gansauge & Meyer 2013), as both methods have been proposed for highly degraded samples.

### Illumina sequencing library preparation

**Figure 2:**
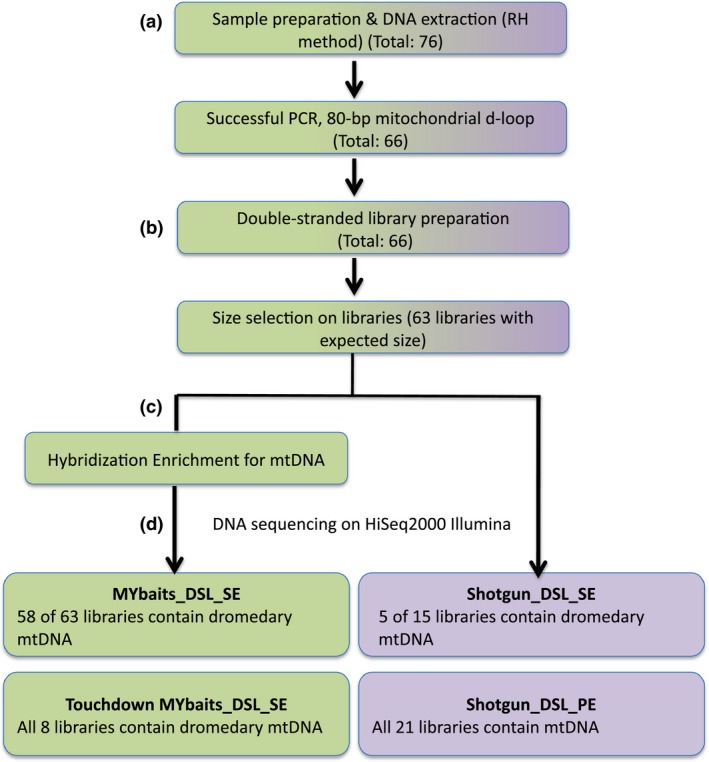
Basic workflow illustrating different steps prior to Illumina sequencing. Summary of the results for enrichment hybridization and shotgun sequencing is shown. [Colour figure can be viewed at wileyonlinelibrary.com]

The quality of DNA extraction in each batch (12 bone samples and two blanks per batch) was evaluated by amplification of an 80‐bp (base pair) fragment (including primers) of the dromedary mtDNA d‐loop (see Appendix S1, Supporting information). Only a subset of ancient‐domestic samples with successful PCR amplification (44 of 54 samples) was further used for library construction and NGS sequencing, while all 22 wild dromedary DNA extracts regardless of positive/negative PCR results were included in further analyses (Fig. 2). The Illumina DSLs were built directly from the DNA extracts as well as extraction blanks and negative controls (library blanks), following the Fortes & Paijmans (2015) protocol. This protocol is based on the original Illumina library construction method by Meyer & Kircher (2010) with specific optimizations for samples with degraded DNA. Purification steps throughout the library construction protocol were performed with MinElute purification columns (Qiagen) according to the manufacturer's instructions. The libraries were constructed using an 8‐bp barcode on the 3′ end of the P5 adapter (directly adjacent to the 5′ end of the aDNA template), which served as an additional means to assign sequences to samples (Fortes & Paijmans 2015). In addition, it provided extra information to filter chimeric reads (or jumping PCR) from the data set and thus increased the confidence in assigning the reads to a particular library. This barcoding method did not require an additional sequence read; the 8‐bp P5 barcode was retrieved as part of the R1 forward reads. The 8‐bp P5 barcode for each sample was identical to its P7 index; sequences of the indices and the modified Illumina adapters are listed in Tables S1 and S2 (Supporting information), respectively.

Following library construction and preindexing amplification, we performed parallel indexing PCRs (to apply the P5 barcode) to maintain more complexity of each library during amplification (see Appendix S1, Supporting information). As endogenous DNA in ancient samples is usually present in low quantity, amplification of the library can introduce biases by amplifying certain fragments. We reduced this loss of complexity by amplifying each library in six parallel indexing PCRs (to apply the P5 barcode), each containing a unique subset of the original library as starting templates (see Appendix S1, Supporting information; library preparation and indexing PCR to apply the P5 barcode). The PCR products were pooled in equimolar ratios, purified through a single Qiagen MinElute spin column and eluted in 20 μL elution buffer (EB) following 10‐min incubation at room temperature. The DSL preparation was performed in a dedicated aDNA laboratory at the University of York, UK, following the standard contamination precautions (Knapp *et al*. 2012). In addition, we constructed seven single‐stranded libraries (SSL) (Gansauge & Meyer 2013) from six wild dromedaries and one giant one‐humped camel (*C. thomasi*) in the presence of one extraction and one library blank (Table S1, Supporting information). The SSL preparations were conducted in a dedicated aDNA laboratory at the University of Copenhagen, Denmark.

### In‐solution hybridization capture and sequencing

Dromedary complete mtDNA was enriched in indexed DSLs (domestic and wild) by in‐solution hybridization capture (Table S3, Supporting information), using MYcroarray's MYbaits kit according to the manufacturer's instructions. We also performed the alternative ‘MYbaits‐touchdown’ (TD) method (Li *et al*. 2013) on DSLs from four domestic and four wild dromedary samples (see Supporting information Table S3; Fig. 2). The hybridization conditions for MYbaits capture were 65 °C for 36 h, vs. 48 h for the MYbaits‐touchdown method with the temperature decreasing from 65 to 50 °C. Following the capture enrichment, 2–4 μL of the indexed libraries was quantified on an agilent bioanalyzer 2100 (software version 1.03). The indexing PCRs (to apply the P5 barcode), in‐solution hybridization enrichment and postcapture amplification were performed in a molecular laboratory at the University of York. The TD hybridization method and the respective postcapture amplification were performed at the Vetmeduni in Vienna, Austria. Among the 66 prepared indexed DSLs, the expected product size of 150–300 bp for three libraries (two ancient‐domestic and one wild) was not detected on 1.5% agarose gel; therefore, these samples were excluded from further analysis (Fig. 2).

Initially, 63 enriched indexed libraries and two library blanks were pooled in equimolar concentrations and single‐end (SE)‐sequenced (read length 100 bp) on one lane of the HiSeq2000 Illumina platform (National High‐throughput DNA Sequencing Centre, University of Copenhagen, Denmark). In another attempt, only indexed libraries from wild samples (21 libraries) were paired‐end (PE)‐shotgun‐sequenced (read length 100 bp) on 1/16 of an Illumina platform lane (Beijing Genomic Institute, China). We also SE‐sequenced a set of 23 indexed libraries (15 shotgun and 8 TD enriched) on another 1/16 of an Illumina platform lane (Beijing Genomic Institute, China).

### Data processing and mapping

The raw reads obtained from the sequenced libraries were trimmed for adapter and index/barcode sequences using the software cutadapt v1.2.1 (Martin 2011). During index/barcode trimming, one error in the index sequence was allowed (parameter −e 0.125). The reads were filtered to a minimum phred‐scaled quality score of 20. The individual read collections were then mapped to the dromedary mtDNA reference (GenBank accession no. NC_009849.1), using the Burrows‐Wheeler Alignment v.0.7.3a (Li & Durbin 2009) with the following parameters (‐l 1024 ‐i 0 ‐o 2 ‐n 0.03 ‐t 6) as optimized for aDNA in Schubert *et al*. (2012). Shotgun sequences were additionally mapped to the dromedary reference genome (Wu *et al*. 2014) (GenBank accession no. GCA_000767585.1), using the same parameters as described. PCR duplicates were removed using Picard MarkDuplicates (http://www.picard.sourceforge.net) to avoid the effect of clonality (PCR duplicates) on downstream analysis. In each sample, the consensus and the polymorphic sites were called with agreement threshold of 50% using samtools package v.0.1.19 (Li *et al*. 2009). The assembly was then checked by eye at each informative polymorphic site to identify sequencing reads conflicting with the reference sequence. Only those sites covered by three unique reads with different start and end positions were accepted as true polymorphism.

To authenticate the sequences obtained as endogenous dromedary mtDNA, we ran mapdamage2.0 (Ginolhac *et al*. 2011; Jónsson *et al*. 2013) to identify DNA damage patterns typical for ancient or degraded DNA. The program uses misincorporation patterns, particularly deamination of cytosine to uracil within a Bayesian framework (Briggs *et al*. 2007; Brotherton *et al*. 2007; Krause *et al*. 2010; Sawyer *et al*. 2012). Nucleotide misincorporations, observed as elevated C to T substitution towards sequencing starts (and complementary increased G to A rates towards the end), are considered as indicative of genuine (endogenous) aDNA. Similarly, an excess of purines at the first nucleotide position of the reference preceding the sequencing reads (and complementary, excess of pyrimidines at the first sequence position following the end of the read) is considered as a typical breakage pattern for aDNA. In order to estimate the performance of different methods (in‐solution capture/TD capture and shotgun sequencing) in terms of the percentage of uniquely mapped reads obtained, we performed the Wilcoxon signed rank test.

### Summary statistics and phylogenetic analysis of modern and ancient‐domestic dromedary mtDNA sequences

Analysis of the ancient‐domestic mtDNA sequences, including the number of variable sites and mitochondrial genetic diversity summary statistics as the number of segregating sites (*s*), the number of haplotypes (*h*), haplotype diversity (*H*_d_), nucleotide diversity (π), average number of pairwise nucleotide differences (k), Tajima's D, Fu and Li's *F* test, as well as a mismatch distribution based on the number pairwise nucleotide differences, was completed with the software dnasp v.5 (Librado & Rozas 2009). For comparisons with modern dromedary mitochondrial diversity, we aligned the ancient mtDNA sequences to nine recently sequenced mitochondrial genomes (E. Mohandesan, R. R. Fitak, J. Corander, A. Yadamsuren, B. Chuluunbat, O. Abdelhadi, A. Raziq, P. Nagy, B. Faye, P. A. Burger. unpublished personal communication; GenBank accession numbers are listed in data accessibility section) as well as to the dromedary mitochondrial reference genome (GenBank accession no. NC_009849.1) and estimated the same diversity parameters from the modern sequences only. For the phylogenetic study of modern and ancient‐domestic dromedary sequences, we performed a median‐joining network (MJN) analysis with network 5.0 (Bandelt *et al*. 1999) with default parameters, displaying the parsimonious (shortest) consensus tree. The program modeltest implemented in mega6 (Tamura *et al*. 2013) was used to identify the appropriate substitution model for the mtDNA sequences. A maximum‐likelihood tree with HKY nucleotide substitution model as best‐fitting model based on Bayesian Information Criterion (BIC) was reconstructed from 16 401 bp of mitochondrial sequences from seven ancient‐domestic dromedary and the available reference sequences from domestic Old World camels (*Camelus dromedarius*: GenBank accession no: NC_009849.1, *Camelus bactrianus*: NC_009628.2 and *Camelus ferus*: NC_009629.2), using mega6. Gaps and missing data were treated with partial deletion, and the 95% site coverage cut‐off was used as default. To obtain statistical support for each node, we used the bootstrap resampling procedure with 100 replications.

## Results

### DNA sequencing

In this study, we investigated the success rate of obtaining DNA sequences from ancient dromedary specimens from prehistoric and historic archaeological sites in Turkey, Syria, Jordan and the UAE. We extracted DNA from 54 ancient‐domestic and 22 wild dromedary bone samples, from which we successfully built 63 DSLs, which were enriched for camel mtDNA using the MYbaits kit. Among these libraries, we recovered reads uniquely mapped to dromedary mtDNA for 58 libraries; four libraries (one ancient‐domestic and three wild samples) produced no camel reads (Table S3, Supporting information; Fig. 2). In addition, we applied TD enrichment to eight of 63 DSLs (four ancient‐domestic and four wild samples) and obtained camel mtDNA reads in all of them (Table S3, Supporting information; Fig. 2).

Furthermore, we SE‐/PE‐shotgun‐sequenced 15 (10 ancient‐domestic and five wild) and 21 (wild) DSLs, respectively (Table S3, Supporting information; Fig. 2). Although in SE shotgun sequencing, 10 samples (six domestic and four wild) failed to produce endogenous mtDNA camel reads (Fig. 2), we successfully recovered nuDNA from these libraries. Using PE shotgun sequencing, we recovered both mt/nuDNA from all libraries.

### Endogenous mtDNA content

Sequencing DSLs using both postcapture and shotgun NGS revealed an extremely low endogenous content of mtDNA ranging from 0.0001% to 0.34% and 0.0001% to 0.004%, respectively (Tables 1 and S3, Supporting information). From all successfully sequenced libraries, we obtained a total of 261 961 806 reads, of which 25 721 unique sequence reads were mapped to the dromedary mtDNA reference genome (Table S3, Supporting information). The proportions of raw, trimmed and uniquely mapped reads to dromedary mtDNA for a few samples using MYbaits/‐TD and shotgun sequencing approaches are shown in Figs S1–S3, Supporting information.

**Table 1:** Sample details and the sequencing scheme used for each sample

Sample ID	% Unique mapped reads to *Camelus dromedarius* mt genome			mt genome length (bp)	%mt genome recovered	Average read depth	GenBank accession no.
	MYbaits capture	MYbaits‐TD capture	Shotgun				
AP2	0.123		0.0008	9943	59.7	2.45	KU605058
AP3	0.294	0.175		15 315	92.0	10.63	KU605059
AQ5	0.013			4083	24.5	2.75	KU605067
AQ24	0.011		0.004	5516	33.1	3.56	KU605060
AQ30	0.241	0.088		15 843	95.1	47.10	KU605061
AQ34	0.058		0	12 162	73.0	8.87	KU605062
AQ40	0.346		0.0003	12 422	74.6	19.33	KU605063
AQ46	0.006		0	4143	24.8	1.44	KU605064
AQ48	0.002		0	3829	23.0	1.56	KU605065
AQ49	0.001		0	2850	17.1	1.62	KU605066
Palm152	0.005	0.001		5149	30.9	1.27	KU605068
Palm157*	0.010			10 890	65.4	2.26	KU605069
Palm171*	0.011			7402	44.4	1.82	KU605070
SAG2	0.028	0.046		14 514	87.2	8.48	KU605071
Tel622	0.0001	0.0006	0.0005				
Tel623	0.0002		0.0009				
Also1	0.0003		0.0008				
Also10	0.0007		0.0008				

All the libraries were built using the double‐stranded library (DSL) method and subjected to sequencing both pre‐ and postcapture using MYbaits. The samples with an asterisk were only sequenced postcapture. The percentage and average coverage of the unique reads mapped to the dromedary mitochondrial genome and the total length of the recovered mtDNA for each sample are shown. For the wild samples, the length of the genome is not calculated, as a result of low numbers of reads mapped to the reference genome.

The postcapture mtDNA reads of the ancient‐domestic samples exhibited DNA damage patterns typical of postmortem depurination and cytosine deamination, indicating that the sequence data truly originated from ancient DNA templates (Fig. S4, Supporting information). The damage pattern was not investigated in wild samples due to the fact that too few reads (2–60 reads) could uniquely be mapped to dromedary mtDNA (Table S3, Supporting information). Overall, we recovered 2850–15 843 bp (17–95%) of the mitochondrial genome from the 14 domestic‐ancient dromedaries, with average read depths of 1.27–47.1‐fold for covered regions over the entire genome (Table 1). We obtained short sequence reads (20–100 bp) from ancient‐domestic enriched libraries with mean fragment length of 65 bp (Table S4; Figs S5 and S6, Supporting information).

### Endogenous nuclear DNA content

To exhaustively investigate the endogenous DNA preservation and endogenous DNA in domestic and wild samples, we mapped the shotgun sequences (SE and PE) to the dromedary whole‐genome sequences (WGS; Wu *et al*. 2014) (Table S5, Supporting information). From all 36 shotgun‐sequenced libraries, we obtained a total of 107 007 621 reads, of which 3 735 270 unique sequence reads (3.53%) were mapped to dromedary WGS with average read depths of 1–1.06‐fold for covered regions over the entire dromedary genome (Table S5, Supporting information). These results show that despite the low amount of total endogenous mtDNA (0.00056%) recovered from these samples in shotgun sequencing experiment, there is a greater quantity of nuclear DNA (3.53%) preserved (Tables S3–S5, Supporting information).

### Enrichment performance on DSL

To evaluate the performance of the in‐solution enrichment method (MYbaits), we computed the percentage of the unique reads that were mapped to the dromedary mtDNA reference sequence. We observed a significant increase in the percentage of on‐target mapped reads in ancient‐domestic camels in the captured libraries (range 0.0017–0.1230, mean 0.0785) compared to shotgun‐sequenced libraries (range 0–0.0042, mean 0.0007; Wilcoxon signed rank *P*‐value = 0.01563). For example, in the sample AQ40, the percentage of the uniquely mapped reads increased by three orders of magnitude postcapture (0.00039–0.34%; Table S3, Supporting information). Overall, the capture method increased the percentage of on‐target mapped reads an average of 187‐fold in our data set of seven samples (ancient‐domestic and wild) for which we performed both shotgun and capture approaches (Table 1). In addition, we observed an increase of average 400‐fold enrichment considering only domestic samples (Table 1). It should be noted that this result is based on only three samples, because seven of the 10 domestic samples did not yield a single camel mtDNA read using shotgun sequencing, despite successful recovery of up to 73% of the mitochondrial genome in the capture approach. Overall, our observed enrichment ranges and averages are similar to those detected in other comparative studies (Ávila‐Arcos *et al*. 2015; Paijmans *et al*. 2015).

### Effect of temperature and hybridization time

We explored the effects of temperature and hybridization time by comparing the number of uniquely mapped reads in the MYbaits capture (65 °C, 36 h) and the alternative MYbaits‐TD (65–50 °C, 48 h) in four ancient‐domestic and four wild individuals. In three domestic samples (AP3, AQ30 and Palm152), we observed a decrease in the percentage of unique mapped reads from the total number of mapped reads in the MYbaits‐TD method. For example in AP3, we recovered 0.29% unique mapped reads with the capture method, while in the TD method the percentage decreased to 0.17%. However in the wild sample (Tel622) and one domestic sample (SAG2), we observed a slight increase in the percentage of the mapped reads with the TD method (Table S3, Supporting information). For these five samples, however, differences in the percentage of endogenous DNA recovered using the TD method are not significant (Wilcoxon signed rank test *P*‐value = 0.4375). An increase in the percentage of PCR duplicate reads (measured as the fraction of the total mapped reads that are PCR duplicates) was observed for 80% of the samples used in the TD experiment (Table S6, Supporting information).

### Mitochondrial genetic diversity of modern and ancient‐domestic dromedaries

**Figure 3:**
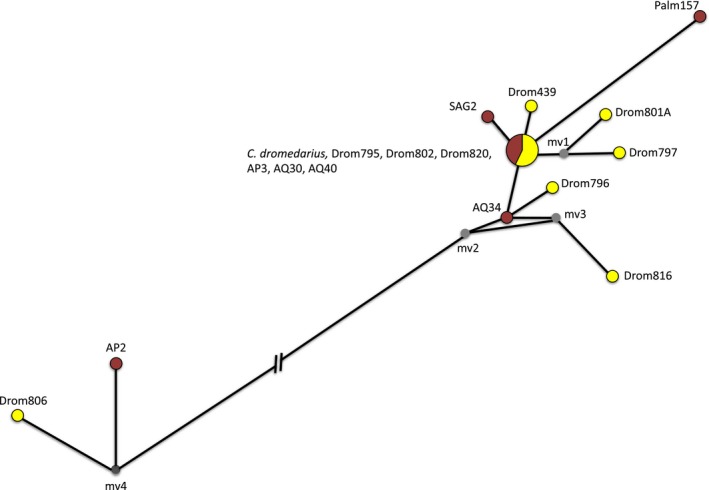
Representation of the mitochondrial haplotypes (6694 bp) retrieved from 10 modern (yellow) and seven ancient (red) domestic dromedaries. Circles are proportional to the sample size. Small grey circles represent median vectors corresponding to missing haplotypes. The genetic distance of 50 fixed polymorphic sites between two haplogroups is not displayed in the graph and is shown with a discontinuous line. [Colour figure can be viewed at wileyonlinelibrary.com]

We obtained 14 partial mitogenomes from ancient‐domestic dromedaries (GenBank accession numbers are listed in data accessibility) with 2850–15 843 bp covered and a mean read depth of 1.27–47.1‐fold (Table 1). Aligning seven ancient‐domestic mtDNA genomes with higher length coverage (59–95%), we obtained 6694 aligned nucleotide sites. These seven ancient samples showed 61 segregating sites with five haplotypes, *H*_d_ of 0.857 and π of 0.00263. In comparison, the 10 modern dromedary sequences (accession numbers for nine genomes are listed in data accessibility) aligned to the same 6694 bp displayed 59 segregating sites, seven haplotypes, *H*_d_ = 0.867 and π = 0.00185 (Table S7, Supporting information). From the ancient‐domestic and modern dromedary mtDNA, we obtained negative values of Tajima's D (−1.69635; *P*‐value < 0.05 and −2.03913; *P*‐value < 0.01) and Fu's and Li's *F* test (−1.96090; *P*‐value < 0.02 and −2.60322; *P*‐value < 0.02), respectively (Table S7, Supporting information). As a test of recent population expansion, we applied mismatch distribution analysis and calculated the observed and expected number of pairwise nucleotide differences in 6694‐bp mtDNA from seven ancient‐domestic and 10 modern dromedaries (Fig. S8, Supporting information). The MJN including modern and ancient‐domestic sequences revealed two haplogroups separated by 50 fixed polymorphic sites, and one haplotype in higher frequency (7/17 samples), and shared between modern and ancient‐domestic samples (Fig. 3). A phylogenetic tree displaying the relationship of the ancient‐domestic mitogenomes with the reference sequences from domestic Old World camels is presented in Fig. S7 (Supporting information). The ancient‐domestic dromedaries and modern dromedary (*Camelus dromedarius*: GenBank accession no. NC_009849.1) cluster together, while the domestic Bactrian camels (*C. bactrianus*: NC_009628.2) and the only remaining wild two‐humped camels (*C. ferus*: NC_009629.2) form a separate sister group.

## Discussion

The ancient‐domestic samples (100 BCE – 1870 CE) used in this study were recovered from sites located in semi‐arid to arid environments, whereas the wild population samples (5000–1400 BCE) originated from hot and partly very humid habitats characterizing the southeast coast of the Arabian Peninsula. Taking into account their archaeological age and the conditions of preservation, we observed a better recovery of endogenous mtDNA from ancient‐domestic dromedary samples in comparison with the wild ones. This is consistent with the observation that arid conditions may be relatively less damaging to DNA than humid conditions even in hot climates (Poinar *et al*. 2003; Haile *et al*. 2009). However, this difference was not observed in the recovery of endogenous nuDNA in the shotgun experiment.

### Effect of temperature and hybridization time on enrichment performance

Despite the use of various target enrichment methods in aDNA research, the efficiency and effectiveness of different hybridization techniques have not yet been fully understood. Paijmans *et al*. (2015) investigated the impact of a key parameter, that is hybridization temperature, on the recovery of mitogenomes from different types of samples (fresh, archival and ancient). They observed better sequence recovery with a constant hybridization temperature of 65 °C in degraded samples, while the touchdown method (65 °C down to 50 °C) yielded the best results for fresh samples. In our study, with a limited sample size (four ancient‐domestic and one wild), we observed no significant effect on the recovery of uniquely mapped reads comparing regular capture and the TD method.

The factors such as hybridization time and binding temperature did not dramatically affect the efficiency of the capture; however, the number of PCR duplicates (clones) increased using the TD method. To obtain adequate amounts of DNA for NGS sequencing, all libraries were amplified 20 cycles during library construction, 10 cycles for indexing and 10–20 cycles postcapture (see Appendix S1, Supporting information). Although the initial DNA concentration used for both capture protocols was the same (>300 ng), the MYbaits‐TD method required an additional 10 cycles of postcapture PCR to generate optimal DNA concentrations for sequencing (Table S6, Supporting information). These additional postcapture PCR cycles may account for the greater sequence clonality observed in the majority of the MYbaits‐TD libraries. At this stage, the reasons underlying the observed differences in capture success are not clear and more data sets and systematic experimental studies are needed to be able to understand the effect of different parameters on capture success.

### Enrichment capture vs. shotgun sequencing in ancient‐domestic samples

We noted a greater recovery (approximately 400‐fold) of endogenous DNA with the capture method for the presumably better preserved ancient‐domestic samples in comparison with shotgun sequencing. This is demonstrated by the recovery of virtually complete mitogenomes from a few ancient‐domestic samples using capture enrichment on just a single sequencing library. This pattern has been observed in other studies where an increase in enrichment of 20–2488‐fold (Paijmans *et al*. 2015) and 6–159‐fold (Carpenter *et al*. 2013) of on‐target content in comparison with shotgun libraries was observed. In addition, the same pattern has been observed by Dabney *et al*. (2013); using shallow shotgun sequencing on a subset of libraries obtained from a Middle Pleistocene cave bear did not recover a single sequence read that aligned with the published Late Pleistocene cave bear mitochondrial genome (Krause *et al*. 2008), while hybridization capture successfully enriched the libraries, aligning with ~4% of the capture reads.

One alternative and cost‐effective approach to enrichment through hybridization is a highly targeted amplicon sequencing technology. Amplicon sequencing allows specifically targeting and deep sequencing multiple regions of interest containing informative genetic variations. This approach reduces the costs and turnaround time where sequencing a large number of samples with high coverage is required. However, in the case of highly degraded samples, most of the fragments are too small for amplification, leaving enrichment through hybridization as method of choice in many studies.

### Enrichment capture vs. shotgun sequencing in wild samples

Our results demonstrate that neither capture nor shotgun methods are efficient in the recovery of mtDNA from wild dromedary samples, whose bones lingered for thousands of years in soils, and which were subjected to varying degrees of humidity and salinity due to fluctuations of the groundwater table. In samples with such low concentration of endogenous DNA, it would be necessary to construct more libraries per sample and to run fewer samples per sequencing lane (cf. Dabney *et al*. 2013; Meyer *et al*. 2014). While this strategy would increase the percentage of endogenous reads, the financial resources in many laboratories preclude this approach.

### Endogenous nuDNA content in ancient‐domestic and wild samples

Mapping the sequence reads obtained from 36 shotgun‐sequenced libraries to the published dromedary genome (Wu *et al*. 2014), we noted a greater recovery of nuDNA (3.53%) in comparison with mtDNA (~0.00056%). We observed that due to the size difference between dromedary mitochondrial (16 Kb) and nuclear genome (2.27 Gb) (Wu *et al*. 2014; Fitak *et al*. 2015), the nuDNA sequence reads outnumber the mtDNA in shotgun sequences. Nevertheless, the data indicate that mt/nuDNA is preserved in our wild samples, and possibly with more DNA extraction and much deeper sequencing for each sample, we would be able to recover more nuDNA from this extinct species.

### Enrichment capture on SSLs in wild samples

Recently, optimized protocols for DNA extraction (Dabney *et al*. 2013) and library preparation (Gansauge & Meyer 2013) have been proposed for highly degraded samples. In particular, the silica‐spin column method proposed in Dabney *et al*. (2013) seems to recover a greater quantity of short DNA fragments, which could significantly enhance the amount of endogenous DNA recovered from archaeological specimens collected in hot environments. The mean fragment length recovered from our ancient‐domestic samples was 65 bp (Table S4; Figs S5 and S6, Supporting information), significantly higher than the fragment length pattern observed in the Sima de los Huesos samples from Spain (Dabney *et al*. 2013). Additional optimization may be obtained using a SSL preparation method (Gansauge & Meyer 2013). Although this method is more costly and time‐consuming, refinements to the SSL construction method may make it more accessible in future (Bennett *et al*. 2014).

We tested the Dabney *et al*.'s (2013) DNA extraction and SSL methods followed by the in‐solution target enrichment on seven wild dromedary camel specimens. However, these methods did not improve the number of obtained DNA sequence reads. This lack of success may be the result of combining these two methods with the capture enrichment. Although the silica‐spin column DNA extraction methods and single‐stranded library protocol are recommended for recovering greater quantities of short DNA fragments, the capture enrichment is generally more efficient on longer fragments. More systematic comparisons of extractions techniques, library building protocols and hybridization capture methodologies will be required in order to optimize the recovery of short ancient DNA templates.

### Mitogenome diversity and demography in ancient‐domestic and modern dromedaries

During the process of domestication, population growth or dispersion of domestic animals across a wider geographical range can be inferred from molecular signals of sudden expansion (Bruford *et al*. 2003). From the mitogenomes of ancient‐domestic and modern dromedaries, we received negative values of Tajima's D and Fu and Li's *F* test (Table S7, Supporting information), respectively, which can indicate demographic expansion assuming the absence of selection. In the MJN (Fig. 3), we observed two haplogroups separated by 50 fixed polymorphisms and a star‐shaped radiation starting from one haplotype in higher frequency, a typical pattern of recent population expansion. Although the mismatch distribution calculated on the number of pairwise differences showed a multimodal distribution related to the two haplogroups, the beginning of the curve is smooth indicative of an expanding population (Fig. S8, Supporting information). Two major haplogroups (H_A_ and H_B_) and signals of population growth in the context of domestication have also been detected in a global sample set of modern dromedary populations (Almathen *et al*. 2016). Comparing mitogenome diversity between ancient‐domestic and modern dromedaries, we observed higher pairwise nucleotide diversity but a slightly lower number of haplotypes and haplotype diversity in the ancient‐domestic dromedary sequences (Table S7, Supporting information). This result can be interpreted as higher retained ancestral diversity in the early‐domestic (ancient) dromedary samples (Troy *et al*. 2001), while in the modern population new haplotypes emerged with only one to two mutational steps (Fig. 3). Evidence for dromedary domestication was found in the southeast coast of the Arabian Peninsula, with a mode of an initial domestication followed by introgression from wild, now‐extinct individuals (Almathen *et al*. 2016).

## Conclusion

The low amount of endogenous sequences in ancient dromedary specimens is an example of the extreme DNA degradation in bone samples from hot and arid environments. Despite the availability of a number of optimized protocols, the recovery of aDNA from poorly preserved samples is still an unresolved issue and hybridization protocols require specific optimization for such specimens. Much deeper sequencing would be necessary; however, this would come at very high costs. This study highlights one of the few successful recoveries of genetic materials from specimens collected from prehistoric and historic archaeological sites located in hot and (hyper)arid environments and reports the first nearly complete mitogenome recovery from ancient‐domestic dromedaries. We also highlight the first recovery of nuDNA from ancient‐domestic and extinct wild dromedary camels.

E.M. performed laboratory work and bioinformatic analyses and wrote the manuscript. C.F.S. performed laboratory work and revised the manuscript. J.P. and B.D.C. provided the samples and revised the manuscript. M.U. and H.P.U. provided the samples. M.H. supported part of the laboratory work and revised the manuscript. P.A.B. managed the project, performed data analysis and wrote the manuscript.

## Data accessibility

The partial mitochondrial genome assemblies from ancient dromedary are archived in GenBank with accession numbers listed below: AP2: KU605058, AP3: KU605059, AQ5: KU605067, AQ24: KU605060, AQ30: KU605061, AQ34: KU605062, AQ40: KU605063, AQ46: KU605064, AQ48: KU605065, AQ49: KU605066, Palm152: KU605068, Palm157: KU605069, Palm171: KU605070, SAG2: KU605071. The complete modern dromedary mitochondrial genomes used for genetic diversity analysis are deposited in GenBank with accession numbers listed below: Drom439 (Qatar, Jordan border): KU605072, Drom795 (Saudi Arabia): KU605073, Drom796 (Saudi Arabia): KU605074, Drom797 (Saudi Arabia): KU605075, Drom801A (Austria): KU605076, Drom802 (UAE, Dubai): KU605077, Drom806 (Kenya): KU605078, Drom816 (Sudan): KU605079, Drom820 (Pakistan): KU605080. In addition, the raw sequence reads from all the libraries sequenced in this study are deposited in Sequence Read Archive under SRA accession: SRP073444 at the National Center for Biotechnology Information (NCBI).

## Supporting information


**Appendix S1**: Supporting Information.
**Table S1** Sample ID, geographical locations and age of ancient‐domestic and wild (extinct) dromedary specimens used in this study.
**Table S2** The Illumina P7 and in‐house designed P5 adapters with two indices, one nested within P7 and one additional 8 bp barcode at 3′ end of P5 adapter.
**Table S3** The number of pre‐processed raw reads, trimmed and uniquely mapped sequence reads to dromedary mtDNA reference genome (GenBank accession no. NC_009849.1) for all DSLs sequenced in this study.
**Table S4** Average sequence read lengths (bp) for enriched DSL (MYbaits/‐TD) for 14 ancient‐domestic and four wild dromedary samples.
**Table S5** The number of pre‐processed raw reads, trimmed and uniquely mapped sequence reads to dromedary whole genome sequences (WGS) (Wu *et al*. 2014) for shotgun‐sequenced libraries from wild dromedary samples.
**Table S6** Percentage of clonal reads (measured as the fraction of the total mapped reads that are PCR duplicates) in MYbaits capture and MYbaits‐TD method.
**Table S7** Genetic diversity of ancient‐domestic and modern dromedaries inferred from 6694 bp mitochondrial DNA data.
**Fig. S1** Proportions of raw (grey), trimmed (blue) and mapped (green) reads obtained from sequencing the DSLs, which were captured with the MYbaits method for 14 ancient‐domestic and four wild dromedary samples.
**Fig. S2** Proportions of raw (grey), trimmed (blue) and mapped (green) reads obtained from sequencing the DSLs, which were captured with MYbaits touchdown approach for four ancient‐domestic and one wild dromedary samples.
**Fig. S3** Proportions of raw (grey), trimmed (blue) and mapped reads (green) obtained from shotgun sequencing of DSL constructed from seven ancient‐domestic and four wild dromedary samples.
**Fig. S4** DNA damage patterns in mitochondrial sequence reads for four ancient‐domestic camel specimens are shown.
**Fig. S5** Average read length distribution for eight ancient‐domestic samples from Jordan (Aqaba) obtained from sequencing the enriched DSLs.
**Fig. S6** Average read length distribution for five ancient‐domestic samples from Syria (Apamea, Palmyra) and one sample from Turkey (Sagalassos) obtained from sequencing the enriched DSLs.
**Fig. S7** The Maximum likelihood phylogenetic tree from complete mitochondrial DNA sequences from Old World camelids.
**Fig. S8** The mismatch distribution of the observed and expected values calculated on the number of pairwise nucleotide differences in 6694 bp mtDNA from seven ancient‐domestic and 10 modern dromedaries.
