# Supplementary material for: Combined hybridization capture and shotgun sequencing for ancient DNA analysis of extinct wild and domestic dromedary camel
Source: Mol Ecol Resour. 2016 Aug 1;17(2):300–13. doi: 10.1111/1755-0998.12551 (PMC5324683; doi:10.1111/1755-0998.12551)
Supplement: Supplementary file 1 — Appendix S1: Supporting Information. Table S1 Sample ID, geographical locations and age of ancient‐domestic and wild (extinct) dromedary specimens used in this study. Table S2 The Illumina P7 and in‐house designed P5 adapters with two indices, one nested within P7 and one additional 8 bp barcode at 3′ end of P5 adapter. Table S3 The number of pre‐processed raw reads, trimmed and uniquely mapped sequence reads to dromedary mtDNA reference genome (GenBank accession no. NC_009849.1) for all DSLs sequenced in this study. Table S4 Average sequence read lengths (bp) for enriched DSL (MYbaits/‐TD) for 14 ancient‐domestic and four wild dromedary samples. Table S5 The number of pre‐processed raw reads, trimmed and uniquely mapped sequence reads to dromedary whole genome sequences (WGS) (Wu et al. 2014) for shotgun‐sequenced libraries from wild dromedary samples. Table S6 Percentage of clonal reads (measured as the fraction of the total mapped reads that are PCR duplicates) in MYbaits capture and MYbaits‐TD method. Table S7 Genetic diversity of ancient‐domestic and modern dromedaries inferred from 6694 bp mitochondrial DNA data. Fig. S1 Proportions of raw (grey), trimmed (blue) and mapped (green) reads obtained from sequencing the DSLs, which were captured with the MYbaits method for 14 ancient‐domestic and four wild dromedary samples. Fig. S2 Proportions of raw (grey), trimmed (blue) and mapped (green) reads obtained from sequencing the DSLs, which were captured with MYbaits touchdown approach for four ancient‐domestic and one wild dromedary samples. Fig. S3 Proportions of raw (grey), trimmed (blue) and mapped reads (green) obtained from shotgun sequencing of DSL constructed from seven ancient‐domestic and four wild dromedary samples. Fig. S4 DNA damage patterns in mitochondrial sequence reads for four ancient‐domestic camel specimens are shown. Fig. S5 Average read length distribution for eight ancient‐domestic samples from Jordan (Aqaba) obtained from sequencing the enrich [file MEN-17-300-s001.docx]

**Supplementary Material**

**Sample collection**

Remains of ancient-domestic dromedary camels were recovered from different archaeological sites in South-West Asia, i.e. Sagalassos Turkey (Turkey, latitude-longitude: 37°40'33.8"N-30°31'12.1"E), Aqaba (Jordan, latitude-longitude: 29°31'18.8"N-35°00'07.8"E), and Apamea (Syria, latitude-longitude: 35°25'24.0"N-36°24'06.2"E) and Palmyra (Syria, latitude-longitude: 34°33'01.9"N-38°16'11.7"E). The osseous remains of wild dromedaries analysed in this study have been collected during excavations at the sites of Al Sufouh-2 (latitude-longitude: 25°7′8.18″N-55°11′0.78″E), Tell Abraq (latitude-longitude: 25.511708N-55.553976E), Al-Buhais 18 (latitude-longitude: 25.048000N-55.725000E), Muweilah (latitude-longitude: 25.3575N-55.3919 E), and Umm-an Nar (latitude-longitude: 29°31′0.12″N, 35°0′0″E); those assigned to the giant wild dromedary (*C. thomasi*) were found in the northern Sudanese Nile Valley (latitude-longitude: 21°47′0″N, 31°22′0″E). The description of the samples and their geographical location are detailed in Table S1 and Fig 1. The wild status of the samples is based on the size of the specimens, together with the archaeological context and dating. Morphology in itself is not decisive, since wild and domestic forms compare very well morphologically.

The ancient-domestic, wild samples from the site Al Sufouh-2 and the *C. thomasi* specimen are housed at the Bavarian State Collection of Anthropology and Palaeoanatomy, Munich (Germany). The wild dromedary samples from Tell Abraq, Al-Buhais 18, Muweilah, and Umm an-Nar were kindly provided by Tübingen University (Germany).

*The Al Sufouh-2 site*

At the site of Al Sufouh-2, more than 17,000 remains of wild dromedaries were unearthed during archaeological excavations in an area located between Dubai city and the Jebel Free Trade zone, 1 km south of the present coastline. The site was in use during the Umm an-Nar and Wadi Suq periods, ca. 2,400-1,400 BCE. Since domestic camels predestined for meat consumption are usually killed and dismembered close to the habitation site, the absence of any traces of architecture (post holes, buildings) near Al Sufouh-2 as well as the fact that most animals excavated were prime-age individuals pertaining to a large-sized population suggest that Al Sufouh-2 was a kill and butchery site of wild dromedaries, used from 2500 – 1500 BCE (von den Driesch *et al.* 2008). These samples were poorly preserved morphologically, many remains being heavily fragmented and showing extensive surface erosion.

*The Tell Abraq site*

Tell Abraq is one of the few ancient sites in South-East Arabia, that contain evidence for a sequence of occupations, which started at about 2,300 BCE during the Umm an-Nar period and lasted for about 2,000 years until the Ed Dur phase around 300 BCE (Potts 1990). The samples from this region are intermediate in size and are from a location in the stratigraphy where both wild and domestic dromedary camel are present. Thus, the sample Tell Abraq 623 is indicated as domestic, but it contains a unique mtDNA (d-loop) haplotype that is not shared with modern dromedary mtDNA haplotypes as shown in Almathen *et al.* (2016), indicating that in fact it is an early domestic or wild specimen. According to Uerpmann and Uerpmann (2002), the Bronze Age (3150 – 1200 BCE) camel remains from Tell Abraq represent a wild population while the samples belonging to the Iron Age (1200 – 586 BCE) are possibly from domestic dromedaries.

*The Umm an-Nar site*

Umm an-Nar is the coastal archaeological site near Abu Dhabi that has become the name-giving site for the Umm an-Nar cultural period in South-East Arabia during 3000-2000 BCE. Based on the archaeological evidence, the wild dromedary was an important game animal in this region during the Early Bronze Age (Uerpmann & Uerpmann 2002).

*The Muweilah site*

Muweilah (Sharjah, UAE) is an archaeological site, which is about 20 km away from both Al Sufouh in the South - West and Tell Abraq in the north. The camel remains of this site that are dated back to Iron Age II (1200 – 586 BCE) and are considered as domestics (Uerpmann & Uerpmann 2002). However, the samples used in this study are older (2000 BCE) than Iron Age II and were morphologically characterized as wild.

*Wadi Halfa region at site 1040*

The *C. thomasi* specimen was found in the early 1960s at site 1040 in the northern Sudanese Nile Valley near Wadi Halfa in the frame of the Combined Nubian Prehistory and Geological Campaign. The animal was found in the top sediment of a 30-meter terrace, under a cover of more recent Nile silt. The top of this terrace has been dated 20,000 BCE (Gautier 1966), but it cannot be entirely excluded that the camel excavated at Site 1040 dates to the much earlier periods of the lower layers of the terrace. Originally believed to exhibit the morphology of a Bactrian camel, it could be shown that the Site 1040 specimen represents a large wild camel with close morphological resemblance to present-day dromedaries (Peters 1998).

**PCR amplification and Sanger sequencing**

We verified the success of DNA extraction and screened for possible contamination by amplifying an 80 bp DNA fragment (including primers) of the camel mitochondrial d-loop region (positions: 15804-15884) from all DNA samples and the extraction blanks. PCR amplifications were carried out in 20 μL volume containing 1x AmpliTaq Gold buffer, 4mM MgCl_2_, 1 mg/ml BSA, 0.25 mM mix dNTPs, 1.5 μM of each primer, 0.5 U/μl of AmpliTaq Gold and 5μL DNA template. PCR mixes were amplified using an iCycler™ Thermal cycler (Bio-RAD) outside the ancient DNA lab. The amplification programme consisted of initial denaturation at 94°C for 9 min followed by 60 cycles consisting of 94°C for 20 sec, 52°C for 30 sec, 72°C for 30 sec and a final extension of 72°C for 4 min. Successful PCR products were purified using the MinElute PCR purification kit (Qiagen’s instruction) and sequenced in both directions on an ABI 3730 XL-Analyzer (Eurofins MWG GmbH, Ebersberg, Germany). The following primers were designed using Primer3 v2.3.6 (Untergasser et al. 2012): Camel_F9 (5'-3'): GGACCATCTCACCTAAAATCG and Camel_R9 (5'-3'): GGCATGGGCTGATTAGTCATT

**Library preparation and indexing PCR**

DNA extracts were built directly into double stranded DNA Illumina libraries (DSL) following the Meyer and Kircher (2010) protocol with some modifications as described in Fortes and Paijmans (2015). Prior to indexing PCR (to apply the P5 barcode), each library was amplified in six parallel amplifications in 20 μL reaction volume each containing 1x AmpliTaq Gold buffer, 2mM MgCl_2_, 0.1 mg/ml BSA, 0.25 mM dNTPs, 0.75 μM of each primary library amplification primer (IS7 and IS8) from Meyer and Kircher (2010), 0.05 U/μL AmpliTaq Gold and 3 μL library template. The amplification programme consisted of initial denaturation at 94°C for 10 min followed by 20 cycles consisting of 94°C for 30 sec, 60°C for 45 sec, 72°C for 45 sec and a final extension of 72°C for 5 min. The amplified libraries for each sample were pooled and purified using the MinElute purification kit according to the manufacturer’s instruction (Qiagen). Following this step, each library was subsequently re-amplified in six parallel indexing PCR (to apply the P5 barcode) using the primers IS4 (Meyer & Kircher 2010) and the respective indexing primers (Table S1). The indexing PCR was carried out in 20 μL reaction volume each containing 3 μL library template with the same PCR master mix set-up and temperature profile, but only for 10 cycles. The double-indexed libraries were purified using the MinElute purification kit (Qiagen) and eluted in 20 μL elution buffer (EB) buffer.

**In-solution capture enrichment and post capture amplification**

We performed the capture enrichment for the entire dromedary mtDNA using 827 unique 80 bp custom designed baits that were tiled every 20 bp (4x tiling). The baits were designed based on whole mitochondrial genome of dromedary. Each library was subjected to in-solution capture hybridization using Mycroarray’s MYbaits kit (according to the manufacturer’s instructions) with some minor modifications as described below: the hybridization master mix was prepared in 18.4 μL instead of 36.8 μL and the capture probes were diluted 1:10 prior to use. Following the capture procedure, the entire enriched libraries were amplified in 40 μL reaction volume containing 1x AmpliTaq Gold buffer, 2mM MgCl_2_, 0.1 mg/ml BSA, 0.25 mM dNTPs, 0.75 μM of each primary library amplification primer (IS5 and IS6) from Meyer and Kircher (2010), 0.05 U/μL AmpliTaq Gold and 20 μL library template. The post capture PCR programme consisted of initial denaturation at 94°C for 10 min followed by 10-20 cycles consisting of 94°C for 30 sec, 60°C for 45 sec, 72°C for 45 sec and a final extension of 72°C for 5 min.

**In-solution touchdown capture enrichment**

For the touchdown hybridization (MYbaits-TD), we followed the method described in (Li *et al.* 2013) with some minor modifications: we performed the touchdown hybridization step for a total of 48 instead of 44 hours, including 12 hours at 65°C, 12 hours at 60°C, 12 hours at 55°C, and 12 hours at 50°C. This step increases the sensitivity to short fragments. After the hybridization step, we added 200 μL MYbaits target enrichment kit binding buffer, which was pre-heated to 48°C (instead of room temperature), to the beads. We then vortexed the tube for 5-10 seconds, placed it on magnetic particle stand for two minutes to pellet the beads and removed and discarded the supernatant. After adding the hybridization solutions to the tubes containing the binding buffer and beads, we incubated them in an oven rotator for 30 min at 48°C instead of room temperature. In theory, performing the binding step at room temperature provides more opportunity for non-specific hybridization and in case of damaged samples with few endogenous templates, possibly decreases the specificity of the binding step (personal communication with MYbaits). The post capture amplification for the touchdown method was performed similar to the in-solution capture enrichment described above. Following the post capture amplification, the indexed libraries were pooled in equimolar ratios and sequenced on the HiSeq2000 Illumina platform.

**References**

Almathen F, Charruau P, Mohandesan E, *et al*. (2016) Dynamics of domestication and trading revealed by the global genetic diversity of the dromedary. *PNAS* doi:10.1073/pnas.1519508113

Briggs AW, Stenzel U, Johnson PL, *et al* (2007) Patterns of damage in genomic DNA sequences from a Neandertal. *Proc Natl Acad Sci U S A*, **104**, 14616-14621.

Dabney J, Knapp M, Glocke I, *et al.* (2013) Complete mitochondrial genome sequence of a Middle Pleistocene cave bear reconstructed from ultrashort DNA fragments. *Proc Natl Acad Sci U S A*, **110**, 15758-15763.

Fortes GG, Paijmans JL (2015) Analysis of Whole Mitogenomes from Ancient Samples. *Methods Mol Biol*, **1347**,179-195.

Gautier A (1966) Camelus thomasi from the northern Sudan and its bearing on the relationship C. thomasi-C. bactrianus. *Journal of Paleontology*, **40**, 1368-1372.

Krause J, Briggs AW, Kircher M, *et al.* (2010) A complete mtDNA genome of an early modern human from Kostenki, Russia. *Curr Biol*, **20**, 231–236.

Li C, Hofreiter M, Straube N, *et al.* (2013) Capturing protein-coding genes across highly divergent species. *Biotechniques*, **54**, 321-326.

Meyer M, Kircher M (2010) Illumina sequencing library preparation for highly multiplexed target capture and sequencing. *Cold Spring Harb Protoc,* doi: 10.1101/pdb.prot5448.

Peters J (1998) Camelus thomasi Pomel, 1893, a possible ancestor of the one-humped camel? *Int. J. Mamm. Biol*, **63**, 372-376.

Potts DT (1990) A Prehistoric Mound in the Emirate of Umm al-Qaiwain, U.A.E. Excavations at Tell Abraq in 1989. Munksgaard, Copenhagen.

Rohland N, Siedel H, Hofreiter M (2010) A rapid column-based ancient DNA extraction method for increased sample throughput. *Mol Ecol Resour*, **10**, 677-683.

Rohland N, Hofreiter M (2007) Comparison and optimization of ancient DNA extraction. *Biotechniques*, **42**, 343-352.

Sawyer S, Krause J, Guschanski K, *et al.* (2012) Temporal patterns of nucleotide misincorporations and DNA fragmentation in ancient DNA. *PLoS ONE*, **7**, e34131.

Uerpmann M, Uerpmann HP (2002) The appearance of the domestic camel in SE-Arabia. *Journal of Oman Studies*, **12**, 235–260.

Untergasser A, Cutcutache I, Koressaar T, *et al*. (2012) Primer3—new capabilities and interfaces. *Nucleic Acids Research*, **40**, e115.

von den Driesch A, Brückner H, Obermaier H, *et al*. (2008) The hunt for wild dromedaries at the United Arab Emirates coast during the 3rd and 2nd millennia BC. Camel bones from the excavations at Al Sufouh 2, Dubai, UAE. In: E. Vila, L. Gourichon, A. M. Choyke & H. Buitenhuis (Eds.), Archaeozoology of the Near East VIII. Proceedings of the eighth international Symposium on the Archaeozoology of southwestern Asia and adjacent areas. Travaux de la Maison de l’Orient et de la Méditerranée, Lyon, **49**, 487-497

Wu H, Guang X, Al-Fageeh MB, *et al.* (2014) Camelid genomes reveal evolution and adaptation to desert environments. *Nat Commun*, **5,** 5188.

**Table S1:** Sample ID, geographical locations and age of ancient-domestic and wild (extinct) dromedary specimens used in this study. The samples with an asterisk (*) are relatively recent (end of the19th -20th century). DNA extraction methods are abbreviated as RH (Rohland & Hofreiter 2007, Rohland *et al*. 2010) and DB (Dabney *et al*. 2013). The success and failure of the methods are indicated as “+” and “-“ respectively. The hybridization enrichment methods are listed as MB (MYbaits) and TD (Mybaits-Touchdown).

| **Sample ID** | **Country**  **Site** | **Find number** | **Excavation year** | **Sample age** | **DNA extraction method / success** | **PCR success rate** | **NGS library / success** | **Hybridization enrichment** | **Whole genome shotgun sequencing (WGSS)** | **Index within P7 adapter** |
| --- | --- | --- | --- | --- | --- | --- | --- | --- | --- | --- |
| **Ancient Domestic Dromedary** | | | | | | | | | | |
| SAG1 | Turkey Sagalassos | SA92N7 | 1992 | Early Byzantine  (450-700 CE) | RH / + | 1/3 | DSL / + | MB |  | TTACTTCT |
| SAG2 | Turkey Sagalassos | SA98PQ35 | 1998 | Early Byzantine  (450-700 CE) | RH / + | 1/1 | DSL / + | MB / TD |  | TAGTTAGG |
| SAG3 | Turkey Sagalassos | SA98LA95 | 1998 | Early Byzantine  (450-700 CE) | RH / + | 1/3 | DSL / + | MB |  | TCATGAAG |
| AP1 | Syria Apamea | G356 | 2007 | Early Byzantine (400-600 CE) | RH / + | 1/2 | DSL / + | MB |  | TCTATTCG |
| AP2 | Syria  Apamea | G357 | 2007 | Early Byzantine (400-600 CE) | RH / + | 1/1 | DSL / + | MB |  | TGGAGTAC |
| AP3 | Syria Apamea | E236 | 2008 | Early Byzantine (400-600 CE) | RH / + | 1/1 | DSL / + | MB / TD |  | TGGCGTTA |
| AP4 | Syria Apamea | E236 | 2008 | Early Byzantine (400-600 CE) | RH / + | 1/3 | DSL / + | MB |  | GATGCCTC |
| AP5 | Syria Apamea | D64 | 2005 | Early Byzantine  (400-600 CE) | RH / + | 1/1 | DSL / + | MB |  | TGGTCCTG |
| AP6 | Syria Apamea | D64 | 2005 | Early Byzantine  (400-600 CE) | RH / + | 1/1 | DSL / + | MB |  | TTAGGATG |
| Palm143 | Syria Palmyra | Pa604/138 | 2002 | 100 BCE- 300 CE | RH / + | 1/3 | DSL / + | MB |  | TTCGTCGG |
| Palm152 | Syria Palmyra | Pa589/123 | 2002 | 100 BCE- 300 CE | RH / + | 1/1 | DSL / + | MB / TD |  | TTGATCCA |
| Palm157 | Syria Palmyra | Pa606/157 | 2002 | 100 BCE- 300 CE | RH / + | 1/1 | DSL / + | MB |  | GAACGCTG |
| Palm164 | Syria Palmyra | Pa606/165 | 2002 | 100 BCE- 300 CE | RH / + | 1/2 | DSL / + | MB |  | GGTACCAA |
| Palm166 | Syria Palmyra | Pa606/607/99 | 2002 | 100 BCE- 300 CE | RH / + | 1/1 | DSL / + | MB |  | GACGTACG |
| Palm171 | Syria Palmyra | Pa607/107 | 2002 | 100 BCE- 300 CE | RH / + | 1/1 | DSL / + | MB |  | GCCAGGTT |
| Palm178 | Syria Palmyra | Pa814/816/178 | 2004 | 100 BCE- 300 CE | RH / + | 1/3 | DSL / + | MB |  | GCGATACG |
| Palm183 | Syria Palmyra | Pa827/176 | 2006 | 100 BCE- 300 CE | RH / + | 1/2 | DSL / + | MB |  | GCTGGTCA |
| AQ1 | Jordan Aqaba | Z1-1 | 2006 | Ottoman (1456-1870 CE) | RH / + | 1/2 | DSL / + | MB |  | GAACCGTT |
| AQ2 | Jordan Aqaba | T50-42 | 2008 | Ottoman (1456-1870 CE) | RH / + | 1/1 | DSL / + | MB |  | GCTTCTCC |
| AQ3 | Jordan Aqaba | D5B-6 | 2007 | Ottoman (1456-1870 CE) | RH / + | 1/2 | DSL / + | MB |  | GCTCTGCT |
| AQ5 | Jordan Aqaba | D5A-1 | 2007 | Ottoman (1456-1870 CE) | RH / + | 1/1 | DSL / + | MB |  | GGTTCAGC |
| AQ8 | Jordan Aqaba | A2-12 | 2007 | Mamluk (1260-1456 CE) | RH / + | 1/3 | DSL / + | MB |  | TCGTTCGA |
| AQ9 | Jordan Aqaba | A2-20 | 2007 | Ottoman (1456-1870 CE) | RH / + | 1/1 | DSL / + | MB |  | GGTAGTTG |
| AQ10 * | Jordan Aqaba | A2-ext-20 | 2007 | end of the19th -20th century | RH / - | 0/3 |  |  |  |  |
| AQ11 * | Jordan Aqaba | A2-ext-21 | 2007 | end of the 19th -20th century | RH / + | 1/1 | DSL / + | MB |  | ATTATCGA |
| AQ12 | Jordan Aqaba | A2-ext-22 | 2007 | Mamluk (1260-1456 CE) | RH / + | 1/1 | DSL / + | MB |  | CAATTGAG |
| AQ13 | Jordan Aqaba | A2-ext-31 | 2007 | Ottoman (1456-1870 CE) | RH / + | 1/1 | DSL / - |  |  | ACCAACCG |
| AQ14 | Jordan Aqaba | A2-ext-35 | 2007 | Ottoman (1456-1870 CE) | RH / + | 1/1 | DSL / + | MB |  | CCATAGTC |
| AQ15 | Jordan Aqaba | A3-1 | 2007 | Ottoman (1456-1870 CE) | RH / - | 0/2 |  |  |  |  |
| AQ16 | Jordan Aqaba | A3-1 | 2007 | Ottoman (1456-1870 CE) | RH / + | 1/1 | DSL / + | MB |  | GTTAAGTT |
| AQ18 | Jordan Aqaba | A3-S182 | 2007 | Mamluk (1260-1456 CE) | RH / - | 0/2 |  |  |  |  |
| AQ19 | Jordan Aqaba | A5-2 | 2007 | Ottoman (1456-1870 CE) | RH / + | 1/2 | DSL / + | MB |  | TCAATGCT |
| AQ20 | Jordan Aqaba | A5-3 | 2007 | Ottoman (1456-1870 CE) | RH / - | 0/3 |  |  |  |  |
| AQ22 | Jordan Aqaba | D2-1 | 2006 | Ottoman (1456-1870 CE) | RH / - | 0/2 |  |  |  |  |
| AQ23 | Jordan Aqaba | D2-3 | 2006 | Ottoman (1456-1870 CE) | RH / + | 1/3 | DSL / + | MB |  | TGGACGCA |
| AQ24 | Jordan Aqaba | D2-11 | 2006 | Mamluk (1260-1456 CE) | RH / + | 1/1 | DSL / + | MB |  | GGAATTGG |
| AQ25 | Jordan Aqaba | D2-16 | 2006 | Mamluk (1260-1456 CE) | RH / + | 1/1 | DSL / + | MB |  | GGTAGAAT |
| AQ26 | Jordan Aqaba | D3-3 | 2006 | Ottoman (1456-1870 CE) | RH / + | 1/1 | DSL / + | MB |  | TGGTATGA |
| AQ27 | Jordan Aqaba | D3-6 | 2006 | Ottoman (1456-1870 CE) | RH / - | 0/3 |  |  |  |  |
| AQ29 | Jordan Aqaba | D3-7 | 2006 | Ottoman (1456-1870 CE) | RH / + | 1/3 | DSL / + | MB |  | AGTTGAAC |
| AQ30 | Jordan Aqaba | D3-14 | 2006 | Mamluk (1260-1456 CE) | RH / + | 1/1 | DSL / + | MB / TD |  | CCTGCCAA |
| AQ32 | Jordan Aqaba | D3-17 | 2006 | Ottoman (1456-1870 CE) | RH / + | 1/1 | DSL / + | MB |  | GTTGCAAC |
| AQ33 | Jordan Aqaba | D3-21 | 2006 | Ottoman (1456-1870 CE) | RH / + | 1/2 | DSL / + | MB |  | TGGTAATT |
| AQ34 | Jordan Aqaba | D3-30 | 2006 | Mamluk (1260-1456 CE) | RH / + | 1/1 | DSL / + | MB |  | GGTTAGTC |
| AQ35 | Jordan Aqaba | D4-1 | 2007 | Mamluk (1260-1456 CE) | RH / + | 1/1 | DSL / - |  |  | TTATCGTC |
| AQ36 | Jordan Aqaba | D4-10 | 2007 | Mamluk (1260-1456 CE) | RH / + | 1/1 | DSL / + | MB |  | CGAATATT |
| AQ40 | Jordan Aqaba | D5A-1 | 2007 | Ottoman (1456-1870 CE) | RH / + | 1/1 | DSL / + | MB |  | CGATCGGA |
| AQ41 | Jordan Aqaba | D5A-2 | 2007 | Ottoman (1456-1870 CE) | RH / - | 0/1 |  |  |  |  |
| AQ44 | Jordan Aqaba | D5A-6 | 2007 | Mamluk (1260-1456 CE) | RH / - | 0/2 |  |  |  |  |
| AQ45 | Jordan Aqaba | D5A-6 | 2007 | Mamluk (1260-1456 CE) | RH / - | 0/2 |  |  |  |  |
| AQ46 | Jordan Aqaba | D5A-7 | 2007 | Mamluk (1260-1456 CE) | RH / + | 1/1 | DSL / + | MB |  | CGTTGCGG |
| AQ47 | Jordan Aqaba | D5B-1 | 2007 | Ottoman (1456-1870 CE) | RH / - | 0/1 |  |  |  |  |
| AQ48 | Jordan Aqaba | D5B-10 | 2007 | Ottoman (1456-1870 CE) | RH / + | 1/1 | DSL / + | MB |  | ATCAAGCA |
| AQ49 | Jordan Aqaba | D5B-11 | 2007 | Mamluk (1260-1456 CE) | RH / + | 1/1 | DSL / + | MB |  | CTTGAGTC |
| **Extinct Wild Dromedary** | | | | | | | | | | |
| Al-Sou-01 | UAE  Al Sufouh2 | A-C7-lvl7 | 2001-2004 | Middle Bronze Age *ca*. 2000-1600 BCE | RH / + | 1/2 | DSL / + | MB | WGSS | GAACGTAT |
| Al-Sou-08 | UAE  Al Sufouh2 | A-C7-lvl5 | 2001-2004 | Middle Bronze Age *ca*. 2000-1600 BCE | RH / - | 0/3 | DSL / - |  |  | GATGATAA |
| Al-Sou-10 | UAE  Al Sufouh2 | A-C7-lvl7 | 2001-2004 | Middle Bronze Age *ca*. 2000-1600 BCE | RH / + | 1/3 | DSL / + | MB | WGSS | GCCTAACT |
| Al-Sou-11 | UAE  Al Sufouh2 | A-1(s)-lvl6 | 2001-2004 | Middle Bronze Age *ca*. 2000-1600 BCE | RH / + | 1/1 | DSL / + | MB / TD | WGSS | GCGTTAGC |
| Al-Sou-12 | UAE  Al Sufouh2 | A-1(s)-lvl6 | 2001-2004 | Middle Bronze Age *ca*. 2000-1600 BCE | RH / +  DB / + | 1/1  1/1 | DSL / +  SSL / + | MB | WGSS | GGCTTGAA |
| Al-Sou-13 | UAE  Al Sufouh2 | A-C7-lvl3 | 2001-2004 | Middle Bronze Age *ca*. 2000-1600 BCE | RH / - | 0/3 | DSL / + | MB | WGSS | GGTCGGCG |
| Al-Sou-21 | UAE  Al Sufouh2 | A-B7-lvl3 | 2001-2004 | Middle Bronze Age *ca*. 2000-1600 BCE | RH / -  DB / + | 0/1  1/1 | DSL / +  SSL / + | MB | WGSS | GTCTTGGC |
| Al-Sou-27 | UAE  Al Sufouh2 | A-C6-lvl5 | 2001-2004 | Middle Bronze Age *ca*. 2000-1600 BCE | RH / - | 0/1 | DSL / + | MB | WGSS | GTTCAATA |
| Al-Sou-29 | UAE  Al Sufouh2 | A-D7-lvl8 | 2001-2004 | Middle Bronze Age *ca*. 2000-1600 BCE | RH / -  DB / + | 0/2  1/1 | +DSL / +  SSL / + | MB | WGSS | TCAGTAGT |
| Al-Sou-31 | UAE  Al Sufouh2 | A-C7-lvl3 | 2001-2004 | Middle Bronze Age *ca*. 2000-1600 BCE | RH / -  DB / + | 0/3  1/2 | DSL / +  SSL / + | MB | WGSS | TGGCGACG |
| Al-Sou-34 | UAE  Al Sufouh2 | A-1N-lvl5 | 2001-2004 | Middle Bronze Age *ca*. 2000-1600 BCE | RH / -  DB / + | 0/2  1/1 | DSL / +  SSL / + | MB | WGSS | TTCGATGA |
| Al-Sou-36 | UAE  Al Sufouh2 | A-C7-lvl4 | 2001-2004 | Middle Bronze Age *ca*. 2000-1600 BCE | RH / +  DB / + | 1/2  1/2 | DSL / +,  SSL / + | MB | WGSS | ATTGATTA |
| Al-Sou-37 | UAE  Al Sufouh2 | A-1-lvl7 | 2001-2004 | Middle Bronze Age *ca*. 2000-1600 BCE | RH / - | 0/3 | DSL / + | MB | WGSS | CCAGCTGG |
| Muw-616 | UAE  Muweilah (Sharjah) | 80 | 2001-2004 | Not known | RH / + | 0/3 | DSL / + | MB | WGSS | CCATATAG |
| Muw-617 | UAE  Muweilah (Sharjah) | 12067 | between 1994 - 2000 | Not known | RH / + | 1/1 | DSL / + | MB | WGSS | CCGATCCT |
| Tell-Ab-618 | UAE  Tell Abraq | 5111 |  | Transition Late Bronze - Early Iron Age  (*ca*. 1260 - 1130 BCE) | RH / + | 1/1 | DSL / + | MB | WGSS | CCTCGACG |
| Muw-619 | UAE  Muweilah (Sharjah) | 99672/26 |  | Not known | RH / - | 0/2 | DSL / + | MB | WGSS | CCTTGAAT |
| BHS-620 | UAE  Al Buhais 18 | 75109 |  | 5000-4000 BCE | RH / - | 0/2 | DSL / + | MB | WGSS | CGACGCCT |
| BHS-621 | UAE  Al Buhais 18 | 60950 | between 1995 - 1998 | 5000-4000 BCE | RH / + | 1/2 | DSL / + | MB / TD | WGSS | CGCGACTC |
| Tell-Ab-622 | UAE  Tell Abraq | 5398 | between 1989- 1993 | Not known | RH / + | 1/3 | DSL / + | MB / TD | WGSS | CTATTCAT |
| Tell-Ab-623 | UAE  Tell Abraq | 5163 |  | Iron Age II  (*ca* 800 - 500 BCE) | RH / + | 1/2 | DSL / + | MB | WGSS | CAACGAGG |
| Um an-Nar-624 | UAE  Umm an-Nar | 1014210 | Between 1994-1997 | Early Bronze Age  (3000-2000 BCE) | RH / + | 1/3 | DSL / + | MB / TD | WGSS | CTTCGTTA |
| **Extinct Giant Wild Dromedary** | | | | | | | | | | |
| *C.thomasi* | Sudan Wadi Halfa region | Site 1040 |  | Upper Palaeolithic, (*ca.* 20,000 BCE) | DB / + | 0/3 | NA  SSL / + | MB |  | TGCATA |

**Table S2:** The Illumina P7 and in-house designed P5 adapters with two indices, one nested within P7 and one additional 8 bp barcode at 3' end of P5 adapter.

|  | **Adapter sequence** |
| --- | --- |
| **P5 (5'-3')** | AATGATACGGCGACCACCGAGATCTACACTCTTTCCCTACACGACGCTCTTCCGATCT_index_ |
| **P7 (5'-3')** | CAAGCAGAAGACGGCATACGAGAT_index_GTGACTGGAGTTCAGACGTGTGCTCTTCCGATCT |

**Table S3:** The number of pre-processed raw reads, trimmed and uniquely mapped sequence reads to dromedary mtDNA reference genome (GenBank accession no. NC_009849.1) for all DSLs sequenced in this study.

| **Sample ID** | **Raw reads** | **Trimmed reads** | **%Trimmed reads** | **Reads used for mapping** | **Mapped reads** | **Unmapped reads** | **PCR duplicates (clones)** | **Unique mapped reads** | **%Unique mapped reads** |
| --- | --- | --- | --- | --- | --- | --- | --- | --- | --- |
| **DSL (MYbaits) single end sequencing** | | | | | | | | | |
| SAG1 | 357298 | 322645 | 90.3 | 336742 | 24 | 336718 | 13 | 11 | 0.003266596 |
| SAG2 | 1834564 | 1351232 | 73.7 | 1832514 | 674 | 1831840 | 150 | 524 | 0.028594597 |
| SAG3 | 2244424 | 1735263 | 77.3 | 2243507 | 41 | 2243466 | 15 | 26 | 0.0011589 |
| AP1 | 1058091 | 917371 | 86.7 | 1056921 | 24 | 1056897 | 16 | 8 | 0.000756916 |
| AP2 | 273532 | 221836 | 81.1 | 267451 | 567 | 266884 | 238 | 329 | 0.123013187 |
| AP3 | 234067 | 196925 | 84.1 | 232166 | 1057 | 231109 | 374 | 683 | 0.294186057 |
| AP4 | 398492 | 123242 | 30.9 | 391494 | 137 | 391357 | 132 | 5 | 0.001277159 |
| AP5 | 28632 | 21335 | 74.5 | 28319 | 0 | 28319 | NA |  | 0 |
| AP6 | 25931 | 17281 | 66.6 | 25348 | 42 | 25306 | 0 | 42 | 0.165693546 |
| Palm143 | 1920986 | 1524655 | 79.4 | 1918439 | 214 | 1918225 | 194 | 20 | 0.001042514 |
| Palm152 | 1383014 | 1099190 | 79.5 | 1382017 | 184 | 1381833 | 110 | 74 | 0.005354493 |
| Palm157 | 1744865 | 1604480 | 92 | 1718217 | 2220 | 1715997 | 2038 | 182 | 0.010592376 |
| Palm164 | 137615 | 114555 | 83.2 | 135774 | 0 | 135774 | NA | 0 | 0 |
| Palm166 | 1458148 | 1372619 | 94.1 | 1446311 | 27 | 1446284 | 12 | 15 | 0.001037121 |
| Palm171 | 1085571 | 967916 | 89.2 | 1061070 | 867 | 1060203 | 748 | 119 | 0.011215094 |
| Palm178 | 1381883 | 1300457 | 94.1 | 1348930 | 8 | 1348922 | 0 | 8 | 0.000593063 |
| Palm183 | 1305315 | 1249569 | 95.7 | 1301054 | 10 | 1301044 | 1 | 9 | 0.000691747 |
| AQ1 | 1038641 | 657609 | 63.3 | 973751 | 200 | 973551 | 173 | 27 | 0.002772783 |
| AQ2 | 47969 | 37959 | 79 | 47762 | 1 | 47761 | 0 | 1 | 0.002093715 |
| AQ3 | 1102542 | 896298 | 81.3 | 1085510 | 57 | 1085453 | 24 | 33 | 0.003040046 |
| AQ5 | 1354066 | 682568 | 50.4 | 1270286 | 290 | 1269996 | 116 | 174 | 0.013697703 |
| AQ8 | 353839 | 280685 | 79.3 | 125851 | 1 | 125850 | 0 | 1 | 0.00079459 |
| AQ9 | 426405 | 370482 | 86.9 | 403331 | 31 | 403300 | 3 | 28 | 0.006942189 |
| AQ11 * | 2881656 | 2765423 | 96 | 2873290 | 77 | 2873213 | 30 | 47 | 0.001635756 |
| AQ12 | 1977823 | 1755526 | 88.8 | 1957596 | 10 | 1957586 | 1 | 9 | 0.000459748 |
| AQ14 | 3679633 | 3052558 | 83 | 3408690 | 1407 | 3407283 | 1381 | 26 | 0.000762756 |
| AQ16 | 5541 | 4252 | 76.7 | 5199 | 1 | 5198 | 0 | 1 | 0.019234468 |
| AQ19 | 1346699 | 1224126 | 90.9 | 1317778 | 4 | 1317774 | 0 | 4 | 0.000303541 |
| AQ23 | 9465 | 6094 | 64.4 | 9420 | 10 | 9410 | 0 | 10 | 0.106157113 |
| AQ24 | 2373186 | 2252646 | 94.9 | 2293212 | 465 | 2292747 | 190 | 275 | 0.011991914 |
| AQ25 | 3035558 | 2956073 | 97.4 | 3024859 | 10 | 3024849 | 1 | 9 | 0.000297535 |
| AQ26 | 16234 | 10 | 0.1 | 16234 | 3 | 16231 | 1 | 2 | 0.012319823 |
| AQ29 | 622283 | 564351 | 90.7 | 608864 | 184 | 608680 | 40 | 144 | 0.023650602 |
| AQ30 | 2356966 | 2249746 | 95.5 | 2344709 | 99486 | 2245223 | 93823 | 5663 | 0.241522509 |
| AQ32 | 884730 | 819954 | 92.7 | 881772 | 715 | 881057 | 662 | 53 | 0.006010624 |
| AQ33 | 1501796 | 1479223 | 98.5 | 1501141 | 16 | 1501125 | 10 | 6 | 0.000399696 |
| AQ34 | 2474866 | 2328253 | 94.1 | 2432729 | 11210 | 2421519 | 9795 | 1415 | 0.058165131 |
| AQ36 | 1513287 | 1417663 | 93.7 | 1508384 | 848 | 1507536 | 525 | 323 | 0.021413645 |
| AQ40 | 1078713 | 1042483 | 96.6 | 1050971 | 30680 | 1020291 | 27042 | 3638 | 0.346156079 |
| AQ46 | 1376325 | 1074021 | 78 | 1360425 | 261 | 1360164 | 172 | 89 | 0.006542073 |
| AQ48 | 4277249 | 3971145 | 92.8 | 4252689 | 474 | 4252215 | 387 | 87 | 0.002045764 |
| AQ49 | 3764895 | 3247392 | 86.3 | 3750078 | 70 | 3750008 | 4 | 66 | 0.001759963 |
| Al-Sou-01 | 2633370 | 1442128 | 54.8 | 2605153 | 12 | 2605141 | 2 | 10 | 0.000383855 |
| Al-Sou-10 | 2953752 | 2062321 | 69.8 | 2951238 | 139 | 2951099 | 116 | 23 | 0.000779334 |
| Al-Sou-11 | 2920140 | 1966206 | 67.3 | 2918925 | 5 | 2918920 | 0 | 5 | 0.000171296 |
| Al-Sou-12 | 3520790 | 2505053 | 71.2 | 3517282 | 30 | 3517252 | 15 | 15 | 0.000426466 |
| Al-Sou-13 | 2845166 | 2063232 | 72.5 | 2840793 | 23 | 2840770 | 14 | 9 | 0.000316813 |
| Al-Sou-21 | 2953568 | 2053422 | 69.5 | 2822752 | 13 | 2822739 | 7 | 6 | 0.000212559 |
| Al-Sou-27 | 3305742 | 1896939 | 57.4 | 3123939 | 20 | 3123919 | 17 | 3 | 9.60326E-05 |
| Al-Sou-29 | 3162183 | 2864356 | 90.6 | 3149152 | 19 | 3149133 | 9 | 10 | 0.000317546 |
| Al-Sou-31 | 1788784 | 1202774 | 67.2 | 1686890 | 8 | 1686882 | 0 | 8 | 0.000474246 |
| Al-Sou-34 | 1660073 | 1324156 | 79.8 | 1648003 | 6 | 1647997 | 0 | 6 | 0.000364077 |
| Al-Sou-36 | 2175536 | 1875067 | 86.2 | 0 | 0 | NA | NA | 0 | 0 |
| Al-Sou-37 | 2057353 | 1899291 | 92.3 | 1948789 | 309 | 1948480 | 277 | 32 | 0.001642045 |
| Muw-616 | 1239566 | 1090107 | 89.9 | 1189806 | 21 | 1189785 | 17 | 4 | 0.000336189 |
| Muw-617 | 2141028 | 2016941 | 94.2 | 2125743 | 2 | 2125741 | 0 | 2 | 9.40848E-05 |
| Tell-Ab-618 | 366303 | 339577 | 92.7 | 357516 | 15 | 357501 | 13 | 2 | 0.000559416 |
| Muw-619 | 2796727 | 2605566 | 93.2 | 0 | 0 | NA | NA | 0 | 0 |
| BHS-620 | 1361857 | 1151704 | 84.6 | 1290342 | 2 | 1290340 | 0 | 2 | 0.000154998 |
| BHS-621 | 1047681 | 754090 | 72 | 908711 | 0 | 908711 | NA | 0 | 0 |
| Tell-Ab-622 | 2947488 | 2040012 | 69.2 | 2232956 | 8 | 2232948 | 4 | 4 | 0.000179135 |
| Tell-Ab-623 | 2018912 | 1817506 | 90 | 1692077 | 4 | 1692073 | 0 | 4 | 0.000236396 |
| Um an-Nar-624 | 2620036 | 2233555 | 85.2 | 2511863 | 2 | 2511861 | 0 | 2 | 7.96222E-05 |
| **DSL (MYbaits-TD) single end sequencing** | | | | | | | | | |
| AP3 | 1129820 | 971217 | 86 | 1108566 | 4763 | 1103803 | 2822 | 1941 | 0.175091064 |
| AQ30 | 10309432 | 9711941 | 94.2 | 10114198 | 286518 | 9827680 | 277566 | 8952 | 0.088509242 |
| Palm152 | 2054415 | 1807530 | 88 | 2025348 | 75 | 2025273 | 40 | 35 | 0.001728098 |
| SAG2 | 2900521 | 2573915 | 88.7 | 2882149 | 4493 | 2877656 | 3164 | 1329 | 0.046111426 |
| Al-Sou-11 | 4069943 | 3446372 | 84.7 | 4056160 | 13 | 4056147 | 2 | 11 | 0.000271192 |
| BHS-621 | 8586370 | 5891331 | 68.6 | 7061053 | 41 | 7061012 | 0 | 41 | 0.00058065 |
| Tell-Ab-622 | 12417856 | 7267654 | 58.5 | 9328663 | 309 | 9328354 | 249 | 60 | 0.000643179 |
| Um an-Nar-624 | 8596978 | 6660391 | 77.5 | 8155315 | 4 | 8155311 | 0 | 4 | 4.90478E-05 |
| **DSL paired end shotgun-sequencing** | | | | | | | | | |
| Al-Sou-01 | 5459006 | 2690008 | 49.27 | 5448561 | 46 | 5448515 | 0 | 46 | 0.00084426 |
| Al-Sou-10 | 4178880 | 2648147 | 63.36 | 4178501 | 36 | 4178465 | 0 | 36 | 0.000861553 |
| Al-Sou-11 | 4700100 | 3182475 | 67.7 | 4700000 | 29 | 4699971 | 0 | 29 | 0.000617021 |
| Al-Sou-12 | 3996628 | 2002971 | 50.1 | 3996360 | 16 | 3996344 | 0 | 16 | 0.00040036 |
| Al-Sou-13 | 3402350 | 2210398 | 64.9 | 3402171 | 19 | 3402152 | 0 | 19 | 0.000558467 |
| Al-Sou-21 | 867776 | 412261 | 47.5 | 866816 | 3 | 866813 | 0 | 3 | 0.000346094 |
| Al-Sou-27 | 5624944 | 2788303 | 49.5 | 5620523 | 15 | 5620508 | 0 | 15 | 0.000266879 |
| Al-Sou-29 | 356474 | 246058 | 69 | 356446 | 2 | 356444 | 0 | 2 | 0.000561095 |
| Al-Sou-31 | 4511580 | 1973116 | 43.7 | 4503928 | 26 | 4503902 | 0 | 26 | 0.000577274 |
| Al-Sou-34 | 5370890 | 2085445 | 38.8 | 5370594 | 21 | 5370573 | 0 | 21 | 0.000391018 |
| Al-Sou-36 | 5852168 | 5126897 | 87.6 | 5849075 | 36 | 5849039 | 0 | 36 | 0.000615482 |
| Al-Sou-37 | 3746720 | 3047748 | 81.3 | 3738528 | 6 | 3738522 | 0 | 6 | 0.000160491 |
| Muw-616 | 1020913 | 307837 | 30.1 | 1020895 | 8 | 1020887 | 4 | 4 | 0.000391813 |
| Muw-617 | 2057968 | 1523444 | 74 | 2057680 | 14 | 2057666 | 4 | 10 | 0.000485984 |
| Tell-Ab-618 | 2857720 | 2334610 | 81.6 | 2855976 | 16 | 2855960 | 6 | 10 | 0.000350143 |
| Muw-619 | 2621618 | 1897258 | 72.3 | 2618297 | 12 | 2618286 | 0 | 12 | 0.000458313 |
| BHS-620 | 1262128 | 852647 | 67.5 | 1258739 | 9 | 1258730 | 0 | 9 | 0.000715001 |
| BHS-621 | 3707396 | 1972936 | 53.2 | 3694517 | 23 | 3694494 | 0 | 23 | 0.000622544 |
| Tell-Ab-622 | 6105296 | 3615947 | 59.2 | 6041387 | 35 | 6041352 | 0 | 35 | 0.000579337 |
| Tell-Ab-623 | 4253344 | 3383104 | 79.53 | 4241168 | 41 | 4241127 | 0 | 40 | 0.000943136 |
| Um an-Nar-624 | 5252284 | 4865248 | 92.6 | 5238509 | 25 | 5238484 | 0 | 25 | 0.000477235 |
| **DSL single end shotgun-sequencing** | | | | | | | | | |
| AP2 | 367288 | 159036 | 43.3 | 367105 | 11 | 367094 | 1 | 10 | 0.002724016 |
| AQ24 | 2664647 | 2461800 | 92.4 | 2651618 | 130 | 2651488 | 17 | 113 | 0.004261549 |
| AQ34 | 2170058 | 1870608 | 86.2 | 2164060 | 0 | 2164060 | NA | 0 | 0 |
| AQ40 | 2284827 | 2125741 | 93 | 2284417 | 9 | 2284408 | 0 | 9 | 0.000393974 |
| AQ46 | 1943014 | 1018165 | 52.4 | 1942168 | 0 | 1942168 | NA | 0 | 0 |
| AQ48 | 2715923 | 2208535 | 81.3 | 2714636 | 0 | 2714636 | NA | 0 | 0 |
| AQ49 | 4486005 | 2906217 | 64.8 | 4484734 | 2 | 4484732 | 0 | 2 | 4.45957E-05 |
| Palm143 | 324015 | 130547 | 40.3 | 323922 | 0 | 323922 | NA | 0 | 0 |
| SAG1 | 41681 | 30973 | 74.3 | 39919 | 0 | 39919 | NA | 0 | 0 |
| SAG3 | 311023 | 140678 | 45.2 | 310935 | 31 | 310904 | 2 | 29 | 0.009326708 |
| Al-Sou-01 | 2678093 | 1153077 | 43.1 | 2667784 | 24 | 2667760 | 10 | 14 | 0.00052478 |
| Al-Sou-10 | 2504877 | 1334034 | 53.3 | 2504303 | 1 | 2504302 | 0 | 1 | 3.99313E-05 |
| Al-Sou-12 | 2223368 | 1303057 | 58.6 | 2223071 | 0 | 2223071 | NA | 0 | 0 |
| Al-Sou-36 | 1854933 | 1561158 | 84.2 | 1853065 | 0 | 1853065 | NA | 0 | 0 |
| Tell-Ab-623 | 3231686 | 2484034 | 76.9 | 3204916 | 0 | 3204916 | NA | 0 | 0 |
| Total number of reads | 261,961,806 |  |  |  |  |  |  | 25,721 |  |

**Table S4:** Average sequence read lengths (bp) for enriched DSL (MYbaits /-TD) for 14 ancient-domestic and four wild dromedary samples.

| **Sample ID** | **Average read length (bp)** | **Standard deviation read length** | |
| --- | --- | --- | --- |
| **Ancient-domestic dromedary** | | | |
| AP2 | 65.71 | | 12.43 |
| AP3 | 62.25 | | 14.93 |
| AQ5 | 62.63 | | 17.39 |
| AQ24 | 73.7 | | 13.56 |
| AQ30 | 64.27 | | 17.47 |
| AQ34 | 65.28 | | 15.74 |
| AQ40 | 60.42 | | 16.78 |
| AQ46 | 64.48 | | 13.07 |
| AQ48 | 65.61 | | 11.60 |
| AQ49 | 69.93 | | 12.90 |
| Palm152 | 67.05 | | 13.96 |
| Palm157 | 66.07 | | 12.48 |
| Palm171 | 67.81 | | 12.17 |
| SAG2 | 64.93 | | 13.56 |
| **Extinct wild dromedary** | | | |
| Tell-Ab-622 | 58.33 | | 32.86 |
| Tell-Ab-623 | 68.5 | | 14.43 |
| Al-Sou-01 | 72.1 | | 14.79 |
| Al-Sou-10 | 46.33 | | 13.52 |

**Table S5:** The number of pre-processed raw reads, trimmed and uniquely mapped sequence reads to dromedary whole genome sequences (WGS) (Wu *et al.* 2014) for shotgun-sequenced libraries from wild dromedary samples.

| **Sample ID** | **Raw reads** | **Trimmed reads** | **%Trimmed reads** | **Reads used for mapping** | **Mapped reads** | **Unmapped reads** | **PCR duplicates (clones)** | **Unique mapped reads** | **%Unique mapped reads** |
| --- | --- | --- | --- | --- | --- | --- | --- | --- | --- |
| **DSL paired end shotgun-sequencing** | | | | | | | | | |
| Al-Sou-01 | 5459006 | 2690008 | 49.27 | 5448561 | 49706 | 5398855 | 3169 | 46537 | 0.854115426 |
| Al-Sou-10 | 4178880 | 2648147 | 63.36 | 4178501 | 45334 | 4133167 | 121 | 45213 | 1.08203875 |
| Al-Sou-11 | 4700100 | 3182475 | 67.7 | 3670016 | 24171 | 3645845 | 19 | 24171 | 0.658089774 |
| Al-Sou-12 | 3996628 | 2002971 | 50.1 | 3996360 | 26353 | 3970007 | 57 | 26296 | 0.657998779 |
| Al-Sou-13 | 3402350 | 2210398 | 64.9 | 3402171 | 31230 | 3370941 | 113 | 31117 | 0.914621869 |
| Al-Sou-21 | 867776 | 412261 | 47.5 | 866816 | 5322 | 861494 | 48 | 5274 | 0.608433624 |
| Al-Sou-27 | 5624944 | 2788303 | 49.5 | 5620523 | 82521 | 5538002 | 12998 | 69523 | 1.236948946 |
| Al-Sou-29 | 356474 | 246058 | 69 | 356446 | 3802 | 352644 | 1 | 3801 | 1.066360683 |
| Al-Sou-31 | 4511580 | 1973116 | 43.7 | 4503928 | 26434 | 4477494 | 775 | 25659 | 0.569702713 |
| Al-Sou-34 | 5370890 | 2085445 | 38.8 | 5370594 | 25868 | 5344726 | 1913 | 23955 | 0.569702713 |
| Al-Sou-36 | 5852168 | 5126897 | 87.6 | 5849075 | 253719 | 5595356 | 2458 | 251261 | 4.29573907 |
| Al-Sou-37 | 3746720 | 3047748 | 81.3 | 3738528 | 55452 | 3683076 | 326 | 55126 | 1.474537572 |
| Muw-616 | 1020913 | 307837 | 30.1 | 1020895 | 5383 | 1015512 | 897 | 4486 | 0.439418354 |
| Muw-617 | 2057968 | 1523444 | 74 | 2057680 | 23471 | 2034209 | 113 | 23358 | 1.13516193 |
| Tell-Ab-618 | 2857720 | 2334610 | 81.6 | 2855976 | 65483 | 2790493 | 1524 | 63959 | 2.239479603 |
| Muw-619 | 2621618 | 1897258 | 72.3 | 2618297 | 59766 | 2558531 | 445 | 59321 | 2.265632967 |
| BHS-620 | 1262128 | 852647 | 67.5 | 1258739 | 9249 | 1249490 | 44 | 9205 | 0.731287423 |
| BHS-621 | 3707396 | 1972936 | 53.2 | 3694517 | 19134 | 3675383 | 521 | 18613 | 0.503800632 |
| Tell-Ab-622 | 6105296 | 3615947 | 59.2 | 6041387 | 75511 | 5965876 | 13145 | 62366 | 1.032312613 |
| Tell-Ab-623 | 4253344 | 3383104 | 79.53 | 4241168 | 148279 | 4092889 | 5992 | 142287 | 3.354901291 |
| Um an-Nar-624 | 5252284 | 4865248 | 92.6 | 5238509 | 221451 | 5017058 | 17775 | 203676 | 3.888052879 |
| **DSL single end shotgun-sequencing** | | | | | | | | | |
| AP2 | 367288 | 159036 | 43.3 | 367105 | 1464 | 365641 | 6 | 1458 | 0.397161575 |
| AQ24 | 2664647 | 2461800 | 92.4 | 2651618 | 85602 | 2566016 | 1584 | 84018 | 3.168555953 |
| AQ34 | 2170058 | 1870608 | 86.2 | 2164060 | 58861 | 2105199 | 534 | 58327 | 2.695257987 |
| AQ40 | 2284827 | 2125741 | 93 | 2284417 | 67749 | 2216668 | 1016 | 66733 | 2.921226729 |
| AQ46 | 1943014 | 1018165 | 52.4 | 1942168 | 2456 | 1939712 | 7 | 2449 | 0.126096198 |
| AQ48 | 2715923 | 2208535 | 81.3 | 2714636 | 35630 | 2679006 | 186 | 35444 | 1.30566308 |
| AQ49 | 4486005 | 2906217 | 64.8 | 4484734 | 44571 | 4440163 | 1874 | 42697 | 0.952052006 |
| Palm143 | 324015 | 130547 | 40.3 | 323922 | 1526 | 322396 | 2 | 1524 | 0.470483635 |
| SAG1 | 41681 | 30973 | 74.3 | 39919 | 565 | 39354 | 42 | 523 | 1.31015306 |
| SAG3 | 311023 | 140678 | 45.2 | 310935 | 1742 | 309193 | 9 | 1733 | 0.557351215 |
| Al-Sou-01 | 2678093 | 1153077 | 43.1 | 2667784 | 15859 | 2651925 | 2147 | 13712 | 0.51398464 |
| Al-Sou-10 | 2504877 | 1334034 | 53.3 | 2504303 | 21621 | 2482682 | 51 | 21570 | 0.8613175 |
| Al-Sou-12 | 2223368 | 1303057 | 58.6 | 2223071 | 11718 | 2211353 | 32 | 11686 | 0.525669221 |
| Al-Sou-36 | 1854933 | 1561158 | 84.2 | 1853065 | 71715 | 1781350 | 476 | 71239 | 3.844387542 |
| Tell-Ab-623 | 3231686 | 2484034 | 76.9 | 3204916 | 86643 | 3118273 | 6543 | 80100 | 2.499285473 |
| Total number of reads | 107,007,621 |  |  |  |  |  |  | 3,735,270 |  |

**Table S6:** Percentage of clonal reads (measured as the fraction of the total mapped reads that are PCR duplicates) in MYbaits capture and MYbaits-TD method. The total number of cycles includes two pre-capture (library construction and indexing PCR to apply the P5 barcode) and one post-capture amplifications.

| **Sample ID** | **Library DNA input (ng)** | **% Clonality** | | **Total number of PCR cycles** | |
| --- | --- | --- | --- | --- | --- |
|  |  | **MYbait** | **MYbait-TD** | **MYbait** | **MYbait-TD** |
| AP3 | 330 | 35.3 | 59.2 | 45 | 55 |
| AQ30 | 624 | 94.3 | 96.8 | 40 | 55 |
| Palm152 | 333 | 59.7 | 53.3 | 40 | 55 |
| SAG2 | 313.2 | 22.25 | 70.4 | 45 | 55 |
| Tell-Ab-622 | 355.5 | 50 | 80.5 | 40 | 55 |

**Table S7**: Genetic diversity of ancient-domestic and modern dromedaries inferred from 6,694 bp mitochondrial DNA data.

| **Sample source** | **Number**  **of samples** | **Variable sites** | **Number**  **of haplotypes** | ***H*d (SD)** | **K** | **θπ (SD)** | **θw (SD)** | **Tajima’s *D*** | **Fu and Li’s F test** |
| --- | --- | --- | --- | --- | --- | --- | --- | --- | --- |
| Ancient-domestic dromedary | 7 | 61 | 5 | 0.857  (0.137) | 17.619 | 0.00263 (0.00146) | 0.00372 (0.00171) | -1.69635 * | -1.96090 ** |
| Modern dromedary | 10 | 59 | 7 | 0.867  (0.107) | 12.400 | 0.00185 (0.00114) | 0.00312 (0.00131) | -2.03913 *** | -2.60322 ** |
| Ancient and  modern dromedary | 17 | 75 | 11 | 0.846 (0,00785) | 13.882 | 0.00207 (0.00091) | 0.00331 (0.00121) | -1.61709 ^n.s^ | -0.91625 ^n.s^ |

*H*d = haplotype diversity, K = average number of nucleotide differences, θπ: theta estimator based on the mean number of nucleotide differences; θw: theta estimator based on the segregating sites; SD: standard deviation values. Statistical significance: **P* <0.05; ***P* <0.02, ****P* <0.01, n.s = not significant.

**Figure S1:** Proportions of raw (grey), trimmed (blue) and mapped (green) reads obtained from sequencing the DSLs, which were captured with the MYbaits method for 14 ancient-domestic and four wild dromedary samples.

**Figure S2:** Proportions of raw (grey), trimmed (blue) and mapped (green) reads obtained from sequencing the DSLs, which were captured with MYbaits touchdown approach for four ancient-domestic and one wild dromedary samples.

**Figure S3:** Proportions of raw (grey), trimmed (blue) and mapped reads (green) obtained from shotgun sequencing of DSL constructed from seven ancient-domestic and four wild dromedary samples.

**Figure S4:** DNA damage patterns in mitochondrial sequence reads for four ancient-domestic camel specimens are shown. For each sample, on top, the base frequencies 5´and 3´ are shown for A, G, C and T for the 10 bases following the break site (grey brackets). Purines (A and G) show an elevated frequency before strand breaks. On the bottom, the C to T nucleotide misincorporation at the first and the last 25 bases of the sequence reads are shown. There is an increase in frequency of T at the 5´and A at the 3´end (G to A misincorporation on the opposite strand of C to T misincorporations), which is a typical pattern for aDNA damage (Sawyer *et al.* 2012, Briggs *et al.* 2007, Krause *et al*. 2010). Y-axis denotes proportion of sites containing a nucleotide change from the reference sequence, and x-axis denotes position along the DNA fragment.

**Figure S5:** Average read length distribution for eight ancient-domestic samples from Jordan (Aqaba) obtained from sequencing the enriched DSLs.

**Figure S6:** Average read length distribution for five ancient-domestic samples from Syria (Apamea, Palmyra) and one sample from Turkey (Sagalassos) obtained from sequencing the enriched DSLs.

**Figure S7:** The Maximum likelihood phylogenetic tree from complete mitochondrial DNA sequences from Old World camelids. The *C. dromedarius* (Genbank Accession no. NC_009849.1), *C. bactrianus* (NC_009628.2), and *C. ferus* (NC_009629.2) sequences were obtained from the NCBI database GenBank. The AP (Apamea; Syria), Palm (Palmyra; Syria) and AQ (Aqaba; Jordan) samples are ancient-domestic dromedary mtDNA obtained in this study. Gaps and missing data were treated with partial deletion and the 95% site coverage cut-off was used as default. The statistical support for each node is based on 100 bootstrap replicates.

**Figure S8:** The mismatch distribution of the observed and expected values calculated on the number of pairwise nucleotide differences in 6,694 bp mtDNA from seven ancient-domestic and 10 modern dromedaries.
